# Enantioseparation of 3-Hydroxycarboxylic Acids via Diastereomeric Salt Formation by 2-Amino-1,2-diphenylethanol (ADPE) and Cinchonidine

**DOI:** 10.3390/molecules28010114

**Published:** 2022-12-23

**Authors:** Srinivas Chandrasekaran, Masaki Tambo, Yuta Yamazaki, Tatsuro Muramatsu, Yusuke Kanda, Takuji Hirose, Koichi Kodama

**Affiliations:** Graduate School of Science and Engineering, Saitama University, 255 Shimo-Okubo, Sakura-Ku, Saitama 338-8570, Japan

**Keywords:** enantioseparation, diastereomeric salt, 3-hydroxycarboxylic acid, chiral recognition, crystal structure

## Abstract

Enantioseparation of 3-hydroxycarboxylic acids via diastereomeric salt formation was demonstrated using 2-amino-1,2-diphenylethanol (ADPE) and cinchonidine as the resolving agents. Racemic 3-hydroxy-4-phenylbutanoic acid (*rac*-**1**), 3-hydroxy-4-(4-chlorophenyl)butanoic acid (*rac*-**2**), and 3-hydroxy-5-phenylpentanoic acid (*rac*-**3**) were efficiently resolved using these resolving agents. Moreover, the successive crystallization of the less-soluble diastereomeric salt of **1** and cinchonidine using EtOH yielded pure (*R*)-**1** · cinchonidine salt in a high yield. The crystal structures of less-soluble diastereomeric salts were elucidated and it was revealed that hydrogen bonding and CH/*π* interactions play an important role in reinforcing the structure of the less-soluble diastereomeric salts.

## 1. Introduction

Chiral molecules are essential in pharmaceuticals, agrochemicals, food supplements, optoelectronic devices, cosmetics, fragrances, flavors, and additives to modify polymer properties [1,2]. However, the presence of an unwanted enantiomer, in the case of drugs such as ethambutol and penicillamine, has adverse side effects [3,4]. This reveals the practical significance of chirality and methods of obtaining enantiopure compounds. Significant advances have been made in asymmetric synthesis [5,6], which generates chiral synthons that are used for synthesizing several drugs [7]. Nevertheless, the preparation of a mixture of two enantiomers (racemate), followed by their enantioseparation (optical resolution), can still be a direct substitute as it is an economical and easy-to-handle process at both laboratory and industrial scales.

The optical resolution of racemates via diastereomeric salt formation is one of the most reliable and frequently used methods for obtaining optically pure acidic and basic compounds [8,9]. In this method, a target racemate is combined with an optically active resolving agent to give a mixture of two diastereomeric salts. When the mixture is recrystallized, the less-soluble diastereomeric salt is preferentially crystallized, and the more-soluble diastereomeric salt remains in the solution owing to their different solubilities. This method offers advantages, such as simple operation, enhancement of the enantiopurity by repeated crystallization, and recyclability of resolving agents, making it an attractive prospect for the pharmaceutical and food industries.

Enantiopure hydroxycarboxylic acids are important compounds that can be widely employed as chiral precursors because their functional groups can be easily modified. Among them, enantiopure 3-hydroxycarboxylic acids have received considerable attention as they have been proven to be valuable synthons and can be used as starting materials in the synthesis of antibiotics, β-amino acids, vitamins, flavors, and pheromones [10,11,12,13]. They are also vital subunits [14] of polyketide natural products, such as amphotericin B [15], tylosin [16], and rosaramicin [17]. Moreover, several enantiopure 3-hydroxycarboxylic acids exhibit critical biological activities, such as antimicrobial and antiviral potential [18,19].

**Figure 1:**
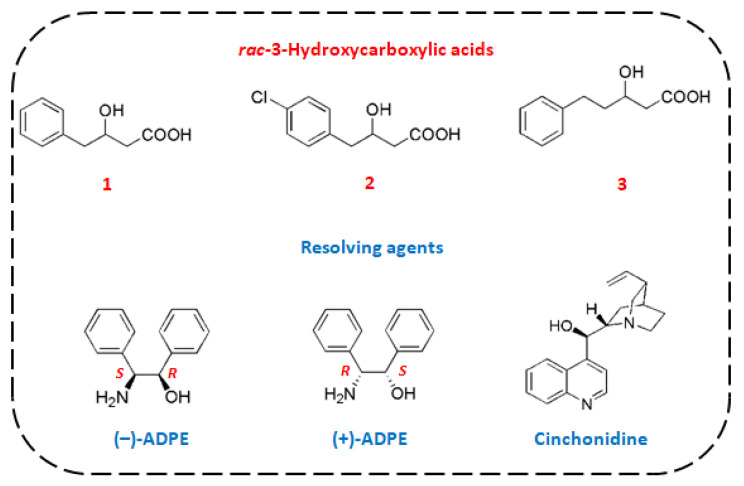
Chemical structures of racemic 3-hydroxycarboxylic acids and resolving agents.

Various chiral amines have been developed as resolving agents for the enantioseparation of carboxylic acids. Previously, we reported the optical resolution of three 3-hydroxypropionic acids with *erythro*-2-amino-1,2-diphenylethanol (ADPE). Crystallographic investigation revealed that the formation of a hydrogen-bonding network and additional CH/*π* interactions play a crucial role in chiral recognition [20,21,22]. In particular, 3-hydroxy-3-phenylpropionic acid was separated in high selectivity with ADPE despite the chiral center is on the remote β-position of the carboxy group. To expand the substrate scope to other β-chiral 3-hydroxycarboxylic acids with a more flexible substituent on the chiral center, we investigated the enantioseparation of racemic 3-hydroxy-4-phenylbutanoic acid (*rac*-**1**), 3-hydroxy-4-(4-chlorophenyl)butanoic acid (*rac*-**2**), and 3-hydroxy-5-phenylpentanoic acid (*rac*-**3**). In these targets, less sterically demanding structure around the chiral center would make their chiral recognition more challenging. As far as we know, the resolution of racemic **1**–**3** via diastereomeric salt formation has never been reported previously. To separate the enantiomers of these acids, cinchonidine, being an inexpensive resolving agent for the resolution of carboxylic acids, was also investigated because it is rigid and bulky and has resolved rather flexible β-chiral 3-hydroxycarboxylic acids [23,24,25]. In this study, we demonstrate the efficient optical resolution of these flexible 3-hydroxycarboxylic acids with ADPE and cinchonidine (Figure 1), and the stability of less-soluble diastereomeric salts is discussed based on their crystal structures.

## 2. Results and Discussion

### 2.1. Optical Resolution of Racemic 3-Hydroxycarboxylic Acids (***1***–***3***) with Enantiopure 2-Amino-1,2-diphenylethanol (ADPE)

The solvents used for crystallization often change the yield and optical purity of the obtained diastereomeric salts. Therefore, the influence of the solvents on the optical resolution of *rac*-**1** with (−)-ADPE was investigated (Table 1). The initial diastereomeric salt mixture was prepared by dissolving equimolar quantities of *rac*-**1** and (−)-ADPE in methanol followed by evaporation. It was then recrystallized from various solvents as described below. The solvents were listed in the order of log P_ow_ as a parameter of polarity. The yield of the deposited salt was calculated based on half the amount of initial salt. A small quantity of the deposited salt was utilized to extract **1**. The recovered **1** was converted to methyl ester and its enantiopurity was determined by HPLC analysis.

In the crystallization of the diastereomeric salt of *rac*-**1** and (−)-ADPE in less polar solvents, CHCl_3_ and THF preferentially afforded the (*R*)-**1** salt as a less-soluble salt with an overall good efficiency (entries 1 and 2). The stereochemical arrangement on the chiral center of **1** in the salt was consistent with that of 3-hydroxy-3-phenylpropionic acid previously studied [22]. In particular, CHCl_3_ afforded the highest efficiency although a large amount of the solvent was necessary. However, crystallization with more polar solvents, AcOEt, 2-PrOH, and aqueous 50% EtOH afforded the (*R*)-**1** salt with moderate to low efficiency (entries 3–5).

The effects of solvents used for recrystallization on the optical resolution of *rac*-**2** with (+)-ADPE (the antipode of (−)-ADPE) were investigated (Table 2). Crystallization of the diastereomeric salt of *rac*-**2** and (+)-ADPE in all the tested solvents afforded the (*S*)-**2** salt with good to high enantiopurity and efficiency. It is worth noting that crystallization with THF produced the (*S*)-**2** salt with high yield and enantiopurity thereby contributing to an exceptionally high efficiency up to 68% (entry 2). Although the structure of the acid **2** is closely related to that of **1**, resolution of *rac*-**2** gave better results than the resolution of *rac*-**1**.

The effects of solvents used for recrystallization during the optical resolution of *rac*-**3** with (−)-ADPE were investigated (Table 3). Crystallization of the diastereomeric salt of *rac*-**3** and (−)-ADPE consistently afforded the (*R*)-**3** salt regardless of the solvents, except in the case of EtOH, which showed no selectivity (entry 6). Although the enantiopurity and resolution efficiency were low in most solvents, less polar solvents afforded the (*R*)-**3** salt with a good enantiopurity and an overall good efficiency (entries 1–3). Despite the fact that **3** has an additional methylene group compared to **1**, the absolute configuration did not change during the resolution of *rac*-**3** with (−)-ADPE, and the (*R*)-**3** salt was obtained. In both the cases, less polar CHCl_3_ afforded the maximum resolution efficiency; however, the value for (*R*)-**3** salt was lower than that of (*R*)-**1**.

### 2.2. Optical Resolution of Racemic 3-Hydroxycarboxylic Acids (***1***–***3***) with Cinchonidine

The effects of solvents used for recrystallization during the optical resolution of *rac*-**1** with cinchonidine were investigated. The experimental procedure was the same as that used for the resolution of **1** with (−)-ADPE (Table 4). Crystallization of the diastereomeric salt of *rac*-**1** and cinchonidine afforded the (*R*)-**1** salt with an overall high efficiency compared to the resolution by (−)-ADPE (Table 1). It was found that polar AcOEt, alcohol solvents, and 1,4-dioxane afforded the (*R*)-**1** salt with high efficiency up to 63% (entries 2–5), whereas less polar THF yielded the (*R*)-**1** salt with only a moderate efficiency (entry 1). In this case, polar solvents afforded the (*R*)-**1** salt efficiently. Such a solvent effect is in contrast with the resolution of *rac*-**1** with (−)-ADPE.

The effects of solvents used for recrystallization during the optical resolution of *rac*-**2** with cinchonidine were investigated (Table 5). Crystallization of the diastereomeric salt of *rac*-**2** and cinchonidine afforded the (*R*)-**2** salt in all the solvents. Polar solvents gave high efficiency (entries 3–6) although less polar CHCl_3_ and THF afforded rather low efficiency (entries 1 and 2). When compared with the resolution results of *rac*-**1**, similar solvent effects were observed for the resolution of *rac*-**2** with cinchonidine, and the (*R*)-**2** salt was obtained. Although 1,4-dioxane yielded good results, it appears ADPE is a more suitable resolving agent for the resolution of *rac*-**2**.

Finally, the effects of solvents used for recrystallization during the optical resolution of *rac*-**3** using cinchonidine were investigated (Table 6). Crystallization of the diastereomeric salt of *rac*-**3** and cinchonidine consistently afforded the (*R*)-**3** salt as less-soluble salt. The absolute configuration did not change, when compared with the resolution of *rac*-**1**. In particular, both the less polar toluene and polar solvents afforded the (*R*)-**3** salt with high efficiency up to 56% (entries 1, 7 and 8). The salt was highly soluble in the examined solvents and only a little amount of EtOH afforded no salt crystal (entry 6).

As far as the solvent effects are concerned, it appears that the solvent polarity influences resolution efficiency to a significant extent. When ADPE was used as the resolving agent, it was almost the case that less polar solvents yielded good results. This is probably due to more effective hydrogen bonds to form the less-soluble salt in less polar solvents. On the other hand, when cinchonidine was used as the resolving agent, more polar solvents have a tendency to yield good results.

Furthermore, in the case of *rac*-**1** and *rac*-**2**, both the resolving agents, ADPE and cinchonidine, have afforded good results (Table 1, Table 2, Table 4 and Table 5). However, in the case of *rac*-**3**, cinchonidine has afforded better resolution results (Table 6) than (−)-ADPE (Table 3). This would be attributed to the smaller structure of ADPE than cinchonidine, which was not suitable for the chiral recognition of longer chain carboxylic acid, *rac*-**3**. On the other hand, cinchonidine is rigid and bulky and, although it is remote from the functional group, it can well distinguish (*R*) or (*S*) in the chiral center, thereby contributing to high resolution efficiency.

### 2.3. Crystallographic Analysis of the Less-Soluble Diastereomeric Salts

Crystallographic investigations were performed to elucidate the structures of less-soluble diastereomeric salts obtained during the optical resolution of **1–3** using ADPE and cinchonidine.

**Figure 2:**
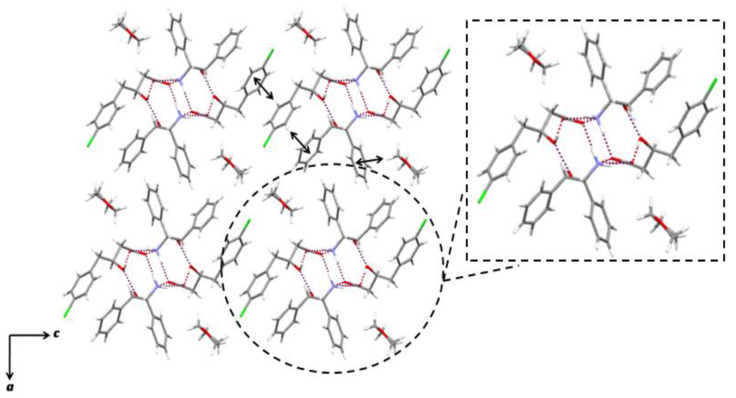
The (*S*)-**2** · (+)-ADPE salt obtained in THF, viewed from the *b*-axis. The dotted lines and arrows indicate hydrogen bonds and CH/*π* interactions, respectively.

The resolution of *rac*-**2** with (+)-ADPE in THF afforded the (*S*)-**2** salt with the highest efficiency (Table 2, entry 2). The structures of the (*S*)-**2** · (+)-ADPE salt crystal obtained in THF are shown in Figure 2. It was revealed that the absolute configuration of **2** was inferred to be (*S*), which was consistent with the resolution results. An array of periodic tubular structures was present along the *b*-axis. A typical columnar hydrogen-bonding network, which was found in other carboxylate salts with enantiopure ADPE [26,27,28], was constructed with a two-fold screw axis (2_1_) from (*S*)-**2** and (+)-ADPE. The ammonium hydrogens of (+)-ADPE were linked to the adjacent carboxylate oxygen atoms of (*S*)-**2** via intermolecular hydrogen bonds. The hydroxy hydrogen of (+)-ADPE was connected to the oxygen of the hydroxy group of (*S*)-**2** via an intermolecular hydrogen bond. The hydroxy hydrogen of (*S*)-**2** was also involved in an intermolecular hydrogen bonding with the carboxylate oxygen of other (*S*)-**2**. It was noteworthy that the crystallization solvent was incorporated in the salt, and the **2**: (+)-ADPE: THF ratio was 1:1:1. The THF molecules were not connected to the tubular structures via hydrogen bonds and remained isolated between the tubular structures to fill the void space. Such an incorporation of cyclic ethers was also be observed for other diastereomeric salts of ADPE and a hydroxycarboxylic acid [29]. In addition to hydrogen bonds, the structure was reinforced by three CH/*π* interactions [30,31], which contributed to its stability. The stereoselectivity of (*S*)-**2** was achieved by fixing its carboxymethyl and hydroxy groups on the stereogenic center by hydrogen bonds as well as by fixing its benzyl group with CH/*π* interactions between the *meta*-CH of (*S*)-**2** and the phenyl group of (+)-ADPE and between the *ortho*-CH of (*S*)-**2** and the phenyl group of other (*S*)-**2**. Such an incorporation of THF in the salt was not observed in the case of **1**, which indicates that the steric effect of the chlorine atom on **2** contributed to create the void space. 

**Figure 3:**
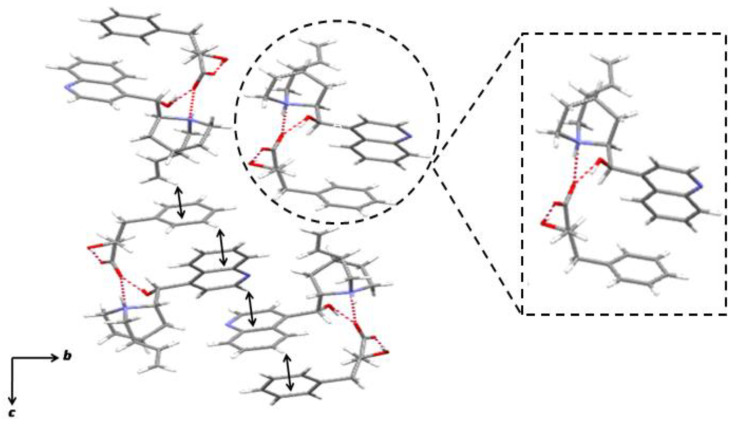
The (*R*)-**1** · cinchonidine salt obtained in an EtOH solution, viewed from the *a*-axis. The dotted lines and arrows indicate hydrogen bonds and CH/*π* interactions, respectively.

The structure of the needle-like crystals (*R*)-**1** · cinchonidine obtained in EtOH is illustrated in Figure 3. The ratio of **1**: cinchonidine was found to be 1:1. The absolute configuration of **1** was inferred to be (*R*), which was consistent with the resolution results (Table 4, entry 4). One carboxylate oxygen of (*R*)-**1**, which points towards the cinchonidine molecule, formed an intermolecular hydrogen bond with the ammonium hydrogen of the azabicyclo[2.2.2]octane group of cinchonidine. The same carboxylate oxygen was held by another intermolecular hydrogen bond that connected it to the hydroxy hydrogen of another cinchonidine. Another carboxylate oxygen of (*R*)-**1**, which points away from the cinchonidine molecule, was also involved in the intermolecular hydrogen bonding with the hydroxy hydrogen of other (*R*)-**1**. Thus, an array of structures with ribbon-like hydrogen-bonding patterns was present along the *a*-axis. These hydrogen-bonding interactions are responsible for reinforcing the crystal structure. The crystal structure was also reinforced by continuous CH/*π* interactions on the aromatic rings of **1** and cinchonidine. The phenyl group of (*R*)-**1** and the quinoline group of cinchonidine were arranged in an edge-to-face orientation. One CH/*π* interaction was present between the CH of the quinoline group of cinchonidine and the quinoline group of other cinchonidine. Three CH/*π* interactions were present on the phenyl group of (*R*)-**1**: one is between the CH of the quinoline group of cinchonidine and the phenyl group of (*R*)-**1**; the other is between the *meta*-CH of (*R*)-**1** and the quinoline group of cinchonidine. Moreover, the CH of the vinyl group of cinchonidine was involved in the CH/*π* interaction with the phenyl group (*R*)-**1**. These CH/*π* interactions were responsible for the recognition of the benzyl group on the chiral center of (*R*)-**1**.

The crystal structure of (*R*)-**2** · cinchonidine salt, which was obtained in EtOH/toluene, is shown in Figure S1. The ratio of **2**: cinchonidine was found to be 1:1. The structure was analogous to that of (*R*)-**1** · cinchonidine despite the presence of a chlorine substituent in **2**, which explains its high efficiency during the resolution of *rac*-**2**.

**Figure 4:**
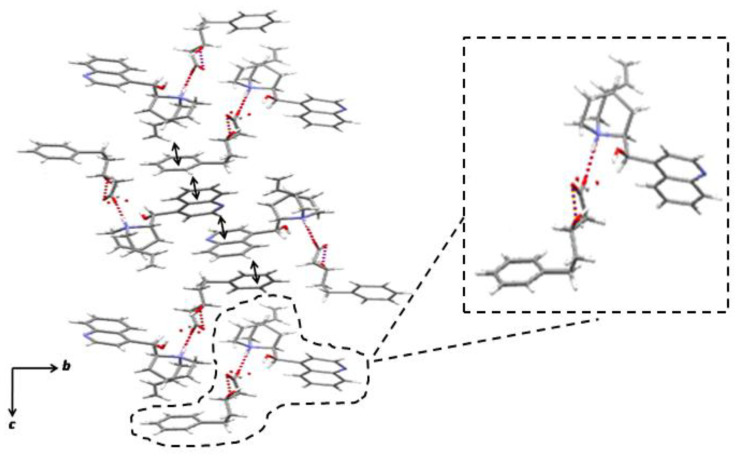
The (*R*)-**3** · cinchonidine salt obtained in an AcOEt solution, viewed from the *a*-axis. The dotted lines and arrows indicate hydrogen bonds and CH/*π* interactions, respectively.

The crystal structure of (*R*)-**3** · cinchonidine salt obtained using AcOEt is illustrated in Figure 4. The ratio of **3**: cinchonidine was found to be 1:1. The absolute configuration of **3** was inferred to be (*R*), which was consistent with the resolution results (Table 6, entry 4). Although the carboxylate moiety of (*R*)-**3** was partly disordered, one carboxylate oxygen of (*R*)-**3**, which points towards the cinchonidine molecule, formed an intermolecular hydrogen bond with the ammonium hydrogen of the azabicyclo[2.2.2]octane group of cinchonidine. Another carboxylate oxygen of (*R*)-**3**, which points away from the cinchonidine molecule, was involved in the intramolecular hydrogen bonding with the hydroxy hydrogen of (*R*)-**3**. There are less intermolecular interactions in (*R*)-**3** · cinchonidine than in the (*R*)-**1** · cinchonidine salt, which probably contributed to its high solubility. They featured ribbon-like networks but only weakly connected along the *a*-axis. Moreover, (*R*)-**3** · cinchonidine exhibited different packing patterns of arrays due to an additional methylene group. The phenyl group of (*R*)-**3** and the quinoline group of cinchonidine were positioned remote to each other. Nevertheless, the crystal structure was reinforced by the same type of continuous CH/*π* interactions as exhibited in (*R*)-**1** · cinchonidine and (*R*)-**2** · cinchonidine. Together with the fixation of carboxyl and hydroxy groups by hydrogen bonds, the terminal phenyl group of (*R*)-**3** was fixed with three kinds of CH/*π* interactions by cinchonidine. Despite its flexibility, *rac*-**3** was efficiently resolved using a large and rigid chiral structure, cinchonidine.

### 2.4. Preparation of the Pure (R)-***1*** Salt with Cinchonidine

The advantage of optical resolution via diastereomeric salt formation is that the optical purity of the less-soluble diastereomeric salt can be enhanced by repeated recrystallization. For example, we have practically demonstrated this phenomenon during the optical resolution of *rac*-**1** with cinchonidine on a larger scale (Table 7).

During the resolution of *rac*-**1** with cinchonidine, EtOH gave remarkable results and afforded the (*R*)-**1** salt efficiently (Table 4, entry 4). Therefore, EtOH was selected as the crystallization solvent to further enhance the purity of the (*R*)-**1** salt via repeated recrystallization. As shown in Table 7, entry 1, the efficiency value after first recrystallization was high, similar to the result obtained in Table 4, entry 4. The enantiopurity of the (*R*)-**1** salt reached more than 99%, with a good yield (49%), thus contributing to the overall good efficiency (49%) after recrystallization from EtOH up to five times (Table 7, entry 5). Therefore, this method can be applied to the production of enantiomerically pure (*R*)-**1** in a larger scale.

## 3. Experimental Section

### 3.1. General Methods

All the reagents and solvents were purchased and used as received. Racemic 3-hydroxycarboxylic acids (*rac*-**1** [32,33], *rac*-**2**, [34] and *rac*-**3** [35,36]) were prepared according to the general procedure and characterized based on the literatures. The enantiomeric excess values were determined by chiral HPLC analyses (Daicel Chiralcel OD-3 column; Eluent: 10% 2-PrOH in hexane; Flow rate: 1.0 mL/min).

### 3.2. Optical Resolution of 3-Hydroxycarboxylic Acids with ADPE/Cinchonidine

The resolution experiment in the case of *rac*-**1** was carried out as follows: Equimolar amounts of *rac*-**1** and (−)-ADPE or cinchonidine were added to a flask and dissolved in methanol followed by evaporation under vacuum. After concentration, the resulting white solid was recrystallized from a suitable solvent by heating to achieve the dissolution of the solid, followed by cooling to room temperature. The obtained crystals were filtered and dried overnight. The apparent yield was calculated based on half the amount of salt. A small portion of the salt was decomposed by the addition of 1N aqueous HCl solution and extracted with diethyl ether. The organic phase was collected and washed with water. Further, it was dried over anhydrous Na_2_SO_4_ and concentrated to obtain **1**. After derivatizing **1** to its corresponding methyl ester by employing TMSCHN_2_ (Trimethylsilyldiazomethane), chiral HPLC analysis was performed.

**1** methyl ester: (Daicel Chiralcel OD-3 column; eluent: 10% 2-PrOH in hexane; flow rate: 1.0 mL/min); *t*_r_(*R*) = 9.1 min, *t*_r_(*S*) = 12.5 min. The absolute configurations were assigned according to the literature [37].

The same procedure has been followed for the optical resolution of *rac*-**2** and *rac*-**3**.

**2** methyl ester: (Daicel Chiralcel OD-3 column; eluent: 10% 2-PrOH in hexane; flow rate: 1.0 mL/min); *t*_r_(*R*) = 8.2 min, *t*_r_(*S*) = 12.2 min. The absolute configurations were deduced from the structural similarity with **1** methyl ester.

**3** methyl ester: (Daicel Chiralcel OD-3 column; eluent: 10% 2-PrOH in hexane; flow rate: 1.0 mL/min); *t*_r_(*S*) = 12.0 min, *t*_r_(*R*) = 13.8 min. The absolute configurations were assigned according to the literature [38].

### 3.3. Single Crystal X-ray Analyses of the Diastereomeric Salt Crystals

Single crystals suitable for X-ray diffraction analysis were prepared by slow evaporation of the saturated solutions of the diastereomeric salts. X-ray crystallographic data were collected on a Bruker Smart APEX II diffractometer with graphite monochromated Mo-Kα radiation. A summary of the diffraction parameters for these structures is shown in Table S1. CCDC 2173286-2173289 contain the supplementary crystallographic data for this paper. These data can be obtained free of charge via http://www.ccdc.cam.ac.uk/conts/retrieving.html, which accessed on 4 December 2022 (or from the CCDC, 12 Union Road, Cambridge CB2 1EZ, UK; Fax: +44 1223 336033; E-mail: deposit@ccdc.cam.ac.uk).

## 4. Conclusions

The enantiomers of 3-hydroxy-4-phenylbutanoic acids (*rac*-**1** and **2**) and 3-hydroxy-5-phenylpentanoic acid (*rac*-**3**) were separated via diastereomeric salt formation with enantiopure 2-amino-1,2-diphenylethanol (ADPE) efficiently, while the resolution of these acids with cinchonidine consistently yielded (*R*)-salts with higher efficiency. The effect of the solvents during recrystallization was discussed based on their polarity. The enhancement of the optical purity of the less-soluble diastereomeric salt by repeated crystallization was demonstrated during the resolution of *rac*-**1** with cinchonidine to obtain pure (*R*)-**1** salt. Crystallographic analysis of the less-soluble diastereomeric salts revealed that hydrogen bonding and CH/*π* interactions played a crucial role in chiral recognition. This study guides the access of enantiomers with simple and economical operations. Further application of this unsophisticated method to other 3-hydroxycarboxylic acids is currently under investigation.

## Figures and Tables

**Table 1:** Optical resolution of racemic 3-hydroxy-4-phenylbutanoic acid (*rac*-**1**) with (−)-ADPE ^a^.

Entry	Recrystallization Solvent (L/mol)	Yield % ^b^	*Ee* % ^c^	Eff. % ^d^
1	CHCl_3_ (152)	86	51 (*R*)	44
2	THF (22)	69	39 (*R*)	27
3	AcOEt (116)	97	10 (*R*)	10
4	2-PrOH (30)	24	30 (*R*)	7
5	50% EtOH (8)	97	21 (*R*)	20

^a^ Equimolar amounts of *rac*-**1** and (−)-ADPE were used. ^b^ The yield was based on half the amount of salt. ^c^ The *ee* was determined by HPLC after derivatization to its corresponding methyl ester. ^d^ Eff. (%) = yield (%) × *ee* (%)/100.

**Table 2:** Optical resolution of racemic 3-hydroxy-4-(4-chlorophenyl)butanoic acid (*rac*-**2**) with (+)-ADPE ^a^.

Entry	Recrystallization Solvent (L/mol)	Yield % ^b^	*Ee* % ^c^	Eff. % ^d^
1	CHCl_3_ (200)	95	38 (*S*)	36
2	THF (8)	90	76 (*S*)	68
3	AcOEt (70)	98	38 (*S*)	37
4	2-PrOH (5)	65	56 (*S*)	37
5	1,4-Dioxane (6)	95	55 (*S*)	52
6	H_2_O (67)	62	49 (*S*)	30

^a^ Equimolar amounts of *rac*-**2** and (+)-ADPE were used. ^b^ The yield was based on half the amount of salt, considering the amount of solvent included. ^c^ The *ee* was determined by HPLC after derivatization to its corresponding methyl ester. ^d^ Eff. (%) = yield (%) × *ee* (%)/100.

**Table 3:** Optical resolution of racemic 3-hydroxy-5-phenylpentanoic acid (*rac*-**3**) with (−)-ADPE ^a^.

Entry	Recrystallization Solvent (L/mol)	Yield % ^b^	*Ee* % ^c^	Eff. % ^d^
1	Toluene (1120)	73	32 (*R*)	23
2	CHCl_3_ (120)	93	34 (*R*)	32
3	THF (18)	65	47 (*R*)	31
4	AcOEt (100)	64	23 (*R*)	15
5	2-PrOH (6)	97	11 (*R*)	11
6	EtOH (10)	92	*Rac.*	0
7	1,4-Dioxane (10)	38	18 (*R*)	7

^a^ Equimolar amounts of *rac*-**3** and (−)-ADPE were used. ^b^ The yield was based on half the amount of salt. ^c^ The *ee* was determined by HPLC after derivatization to its corresponding methyl ester. ^d^ Eff. (%) = yield (%) × *ee* (%)/100.

**Table 4:** Optical resolution of racemic 3-hydroxy-4-phenylbutanoic acid (*rac*-**1**) with cinchonidine ^a^.

Entry	Recrystallization Solvent (L/mol)	Yield % ^b^	*Ee* % ^c^	Eff. % ^d^
1	THF (12)	61	33 (*R*)	20
2	AcOEt (60)	91	59 (*R*)	54
3	2-PrOH (4)	81	61 (*R*)	49
4	EtOH (2)	96	66 (*R*)	63
5	1,4-Dioxane (8)	67	63 (*R*)	42

^a^ Equimolar amounts of *rac*-**1** and cinchonidine were used. ^b^ The yield was based on half the amount of salt. ^c^ The *ee* was determined by HPLC after derivatization to its corresponding methyl ester. ^d^ Eff. (%) = yield (%) × *ee* (%)/100.

**Table 5:** Optical resolution of racemic 3-hydroxy-4-(4-chlorophenyl)butanoic acid (*rac*-**2**) with cinchonidine ^a^.

Entry	Recrystallization Solvent (L/mol)	Yield % ^b^	*Ee* % ^c^	Eff. % ^d^
1	CHCl_3_ (5)	29	59 (*R*)	17
2	THF (6)	28	58 (*R*)	16
3	AcOEt (80)	50	87 (*R*)	44
4	2-PrOH (4)	92	40 (*R*)	37
5	EtOH (4)	67	54 (*R*)	36
6	1,4-Dioxane (15)	91	55 (*R*)	50

^a^ Equimolar amounts of *rac*-**2** and cinchonidine were used. ^b^ The yield was based on half the amount of salt. ^c^ The *ee* was determined by HPLC after derivatization to its corresponding methyl ester. ^d^ Eff. (%) = yield (%) × *ee* (%)/100.

**Table 6:** Optical resolution of racemic 3-hydroxy-5-phenylpentanoic acid (*rac*-**3**) with cinchonidine ^a^.

Entry	Recrystallization Solvent (L/mol)	Yield % ^b^	*Ee* % ^c^	Eff. % ^d^
1	Toluene (2)	98	57 (*R*)	56
2	CHCl_3_ (1)	82	12 (*R*)	10
3	THF (3)	84	45 (*R*)	38
4	AcOEt (9)	98	47 (*R*)	46
5	2-PrOH (1)	68	62 (*R*)	42
6	EtOH (1)	Not crystallized	-	-
7	1,4-Dioxane (1)	99	55 (*R*)	54
8	50% EtOH (2)	87	62 (*R*)	54

^a^ Equimolar amounts of *rac*-**3** and cinchonidine were used. ^b^ The yield was based on half the amount of salt. ^c^ The *ee* was determined by HPLC after derivatization to its corresponding methyl ester. ^d^ Eff. (%) = yield (%) × *ee* (%)/100.

**Table 7:** Successive optical resolution of racemic 3-hydroxy-4-phenylbutanoic acid (*rac*-**1**) with cinchonidine ^a^ using EtOH.

Entry	Recrystallization Solvent (mL)	Number of Times of Crystallization	Yield % ^b^	*Ee* % ^c^	Eff. % ^d^
1	EtOH (14.8)	1	106	60 (*R*)	64
2	EtOH (10.1)	2	83.8	80 (*R*)	67
3	EtOH (11.5)	3	66.3	96 (*R*)	64
4	EtOH (10.8)	4	55.9	98 (*R*)	55
5	EtOH (6.3)	5	49.0	>99 (*R*)	49

^a^ 12.0 mmol *rac*-**1** and cinchonidine were initially used. ^b^ The yield was based on half the amount of initial salt. ^c^ The *ee* was determined by HPLC after derivatization to its corresponding methyl ester. ^d^ Eff. (%) = yield (%) × *ee* (%)/100.

## Data Availability

Data is contained within the article or supplementary material.

## Supplementary Materials

The following supporting information can be downloaded at: https://www.mdpi.com/article/10.3390/molecules28010114/s1, HPLC charts for Table 1, Table 2, Table 3, Table 4, Table 5 and Table 6; Figure S1: The (*R*)-2 · cinchonidine salt obtained in an EtOH/toluene solution, viewed from the *a* axis. The dotted lines and arrows indicate hydrogen bonds and CH/π interactions, respectively; Table S1: Summary of crystallographic data reported in this study.

## Sample Availability

Samples of the compounds are not available from the authors.
