# Supplementary material for: Enantioseparation of 3-Hydroxycarboxylic Acids via Diastereomeric Salt Formation by 2-Amino-1,2-diphenylethanol (ADPE) and Cinchonidine
Source: Molecules. 2022 Dec 23;28(1):114. doi: 10.3390/molecules28010114 (PMC9822485; doi:10.3390/molecules28010114)
Supplement: Supplementary file 1 [file molecules-28-00114-s001.zip › molecules-2109975-supplementary.pdf]

Enantioseparation of 3-hydroxycarboxylic acids *via* diastereomeric salt formation by 2-amino-1,2-diphenylethanol (ADPE) and cinchonidine

Srinivas Chandrasekaran, Masaki Tambo, Yuta Yamazaki, Tatsuro Muramatsu, Yusuke Kanda, Takuji Hirose, Koichi Kodama*

Graduate School of Science and Engineering, Saitama University, 255 Shimo-Okubo, Sakura-Ku, Saitama, 338-8570, Japan

Table of Contents

HPLC charts for Tables 1-6
Figure S1
Table S1

Chromatogram Report

Table 1, Entry 1

分析条件

データファイル名	:	rac-1; (-)-ADPE; CHCl3-12022Y11M07D19h03m43s.crm
解析ファイル名	:	
チャンネルNo.	:	1
分析時間	:	20.0 min
取込間隔	:	200 msec
データ保存場所	:	c:¥users¥有機工業¥documents¥クロマトプロデータ¥srinivas¥second paper¥
分析日時	:	Mon Nov 07 19:03:43 2022
コメント	:	rac-1; (-)-ADPE; CHCl3-1

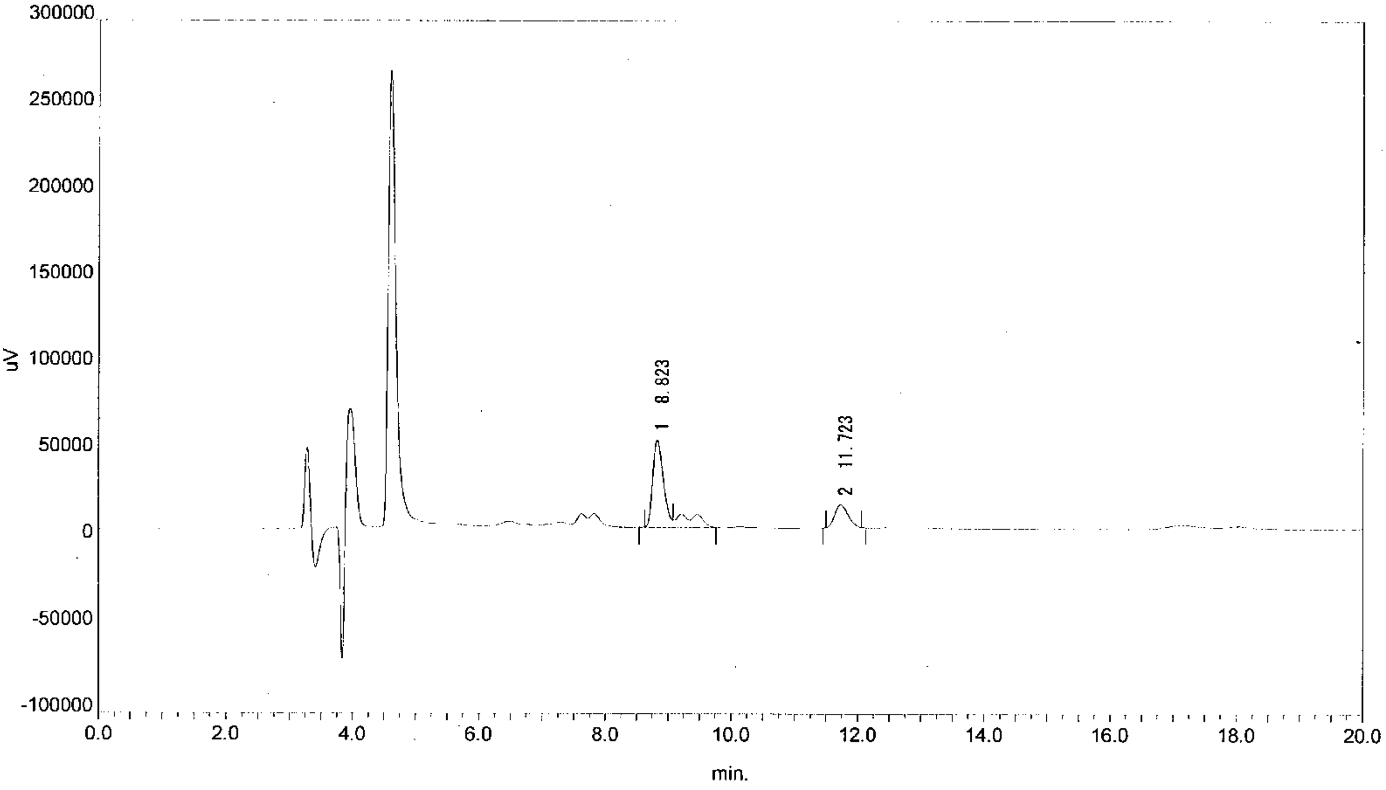

解析結果

No.	Rt(min)	ピーク名	面積	面積(%)	高さ	NTP	対称性	分離度
1	8.82		594094.449	75.2938	50388	11859.7	------	8.018
2	11.72		194941.020	24.7062	13207	13794.2	1.245	------
			789035.470	100.0000	63595			

Chromatogram Report

Table 1, Entry 2

分析条件

データファイル名	: rac-1; (-)-ADPE; AcOEt2022Y11M08D00h37m47s.crm
解析ファイル名	:
チャンネルNo.	: 1
分析時間	: 20.0 min
取込間隔	: 200 msec
データ保存場所	: c:¥users¥有機工業¥documents¥クロマトプロデータ¥srinivas¥second paper¥
分析日時	: Tue Nov 08 00:37:47 2022
コメント	: rac-1; (-)-ADPE; AcOEt

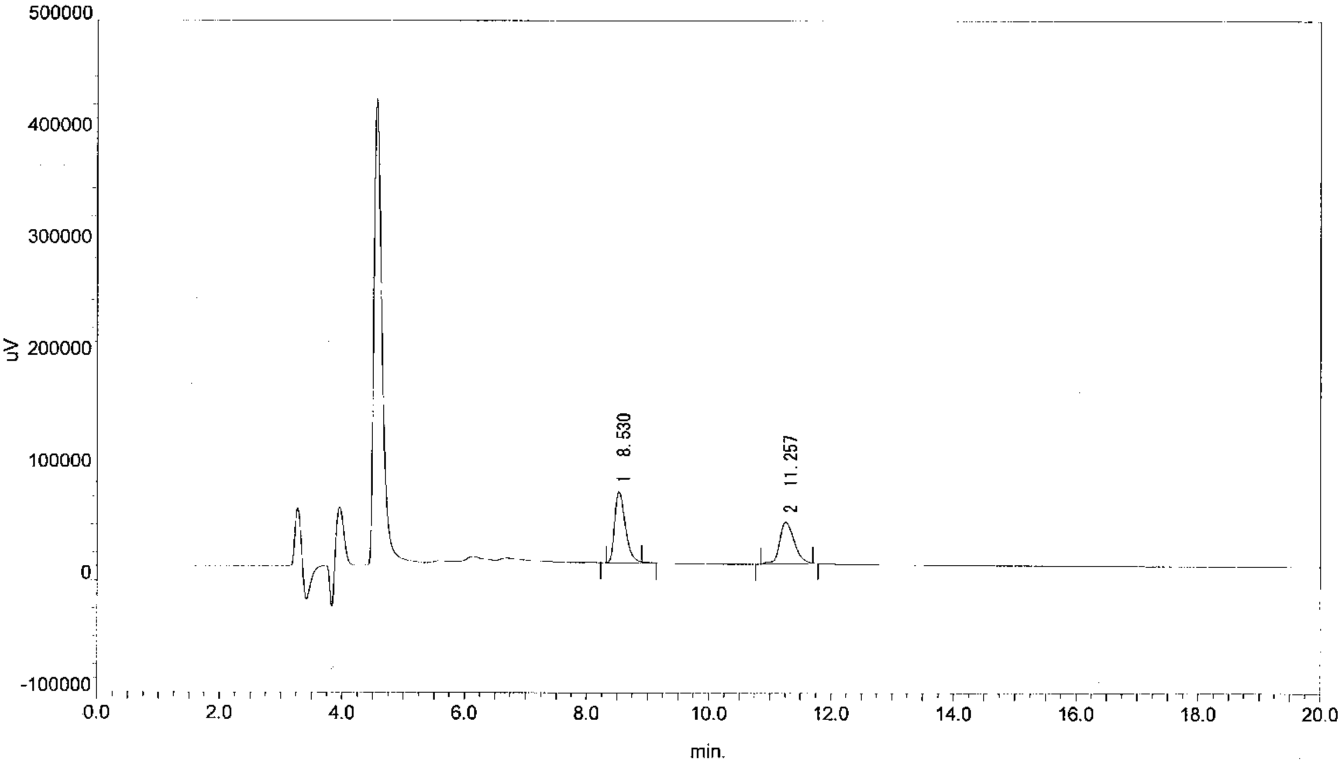

解析結果

No.	Rt(min)	ピーク名	面積	面積(%)	高さ	NTP	対称性	分離度
1	8.53		769079.016	55.2297	63597	10856.3	1.450	7.103
2	11.26		623429.900	44.7703	37374	10460.2	1.224	------
			1392508.916	100.0000	100971			

Chromatogram Report

Table 1, Entry 3

分析条件
データファイル名 : ADPE(THF2回目)2016Y08M30D16h57m28s.crm
解析ファイル名 :
チャンネルNo. : 1
分析時間 : 12.0 min
取込間隔 : 200 msec
データ保存場所 : c:¥users¥有機工業¥documents¥クロマトプロデータ¥yamazaki¥分割¥
分析日時 : Tue Aug 30 16:57:28 2016
コメント : ADPE(THF2回目)

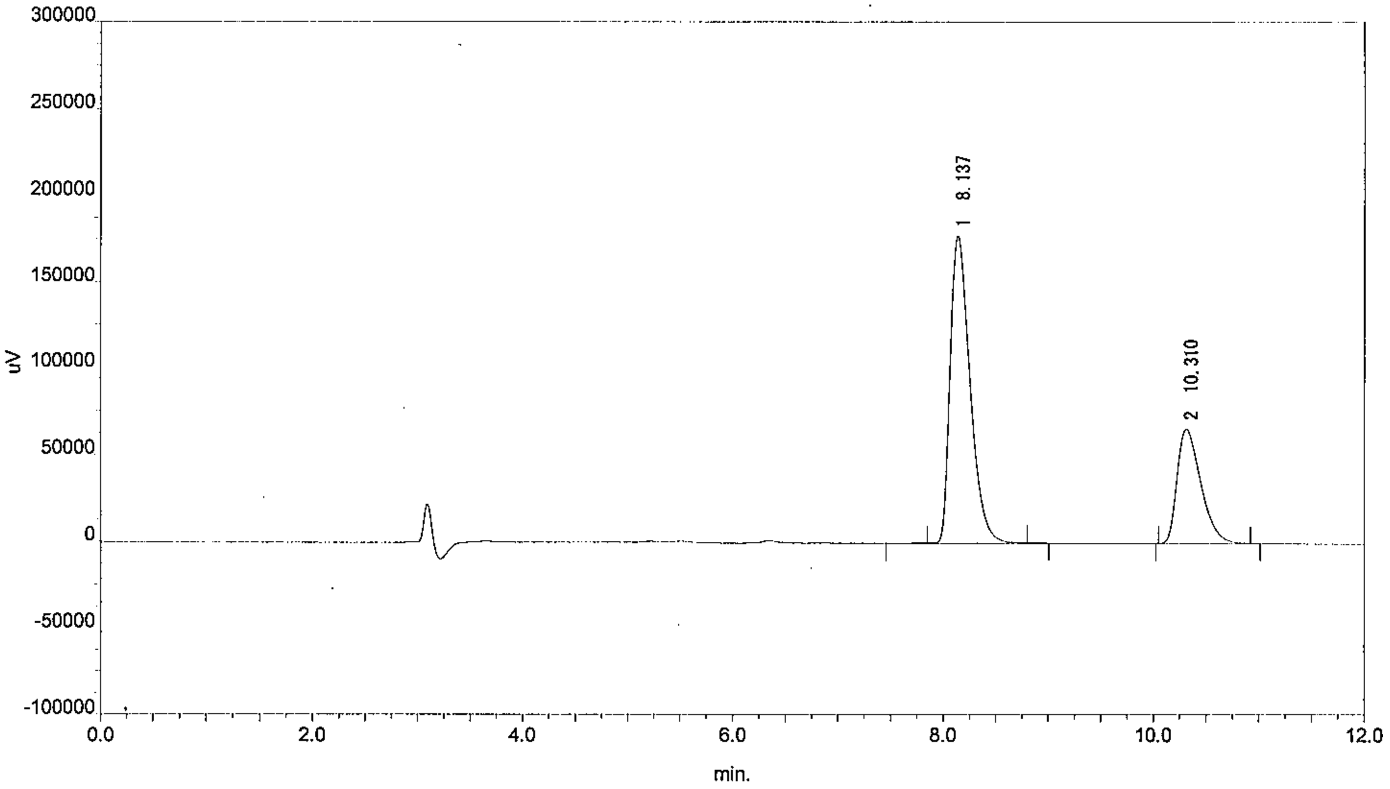

解析結果

No.	Rt(min)	ピーク名	面積	面積(%)	高さ	NTP	対称性	分離度
1	8.14		2338863.282	69.3143	177910	8525.8	1.532	5.616
2	10.31		1035423.588	30.6857	66106	9575.1	1.516	―――
			3374286.869	100.0000	244016			

Chromatogram Report

Table 1, Entry 4

分析条件

データファイル名	:	ADPE (2-PrOH) 2016Y10M08D01h52m38s.crm
解析ファイル名	:	
チャンネルNo.	:	1
分析時間	:	22.7 min
取込間隔	:	200 msec
データ保存場所	:	c:¥users¥有機工業¥documents¥クロマトプロデータ¥yamazaki¥分割¥
分析日時	:	Sat Oct 08 01:52:38 2016
コメント	:	ADPE (2-PrOH)

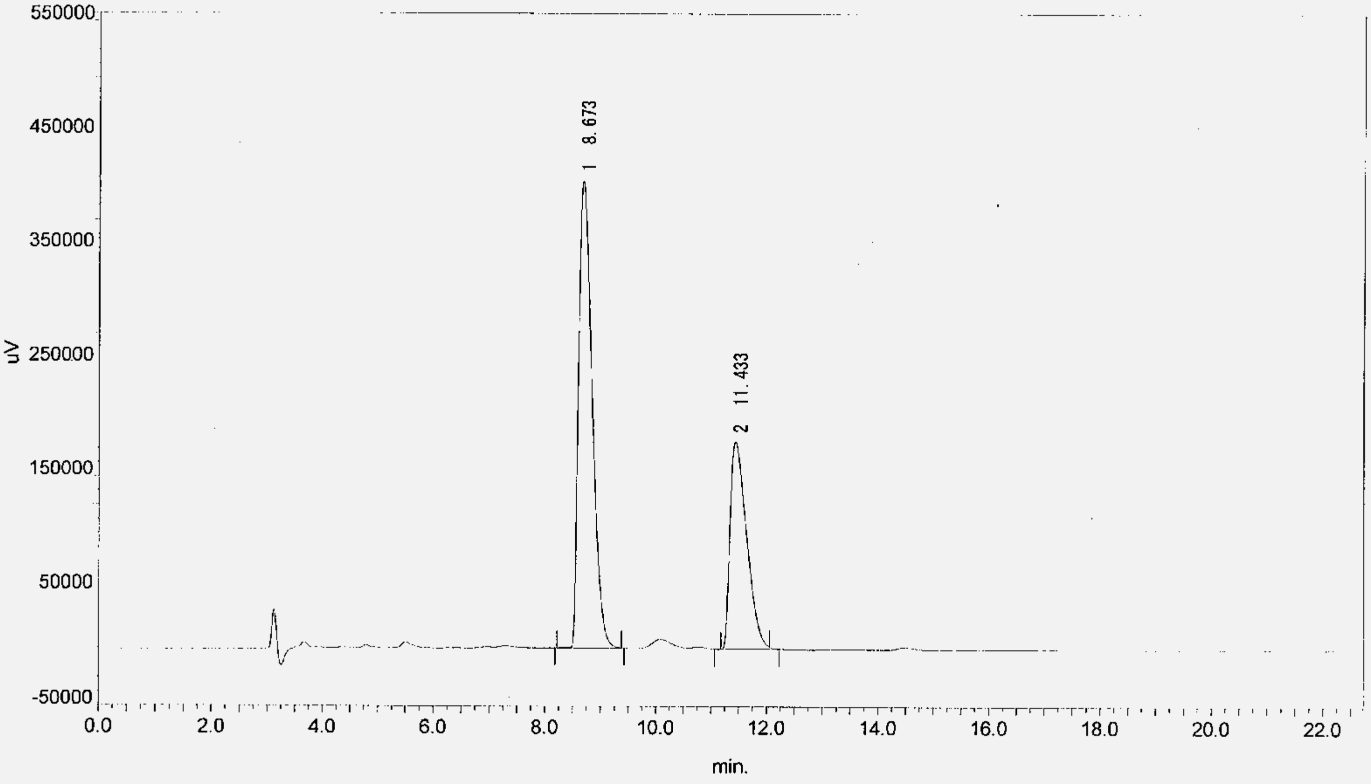

解析結果

No.	Rt(min)	ピーク名	面積	面積(%)	高さ	NTP	対称性	分離度
1	8.67		6946479.600	64.7605	409921	5972.1	1.769	5.486
2	11.43		3779940.520	35.2395	181551	6735.8	1.877	――――
			10726420.120	100.0000	591472			

Chromatogram Report

Table 1, Entry 5

分析条件
データファイル名 : 3-ヒドロキシ-4-フェニルブタン酸メチル(50%EtOH一回目)2016Y07M21D12h43m54s.crm
解析ファイル名 :
チャンネルNo. : 1
分析時間 : 12.9 min
取込間隔 : 200 msec
データ保存場所 : c:¥users¥有機工業¥documents¥クロマトプロデータ¥yamazaki¥分割¥
分析日時 : Thu Jul 21 12:43:54 2016
コメント : 3-ヒドロキシ-4-フェニルブタン酸メチル(50%EtOH一回・

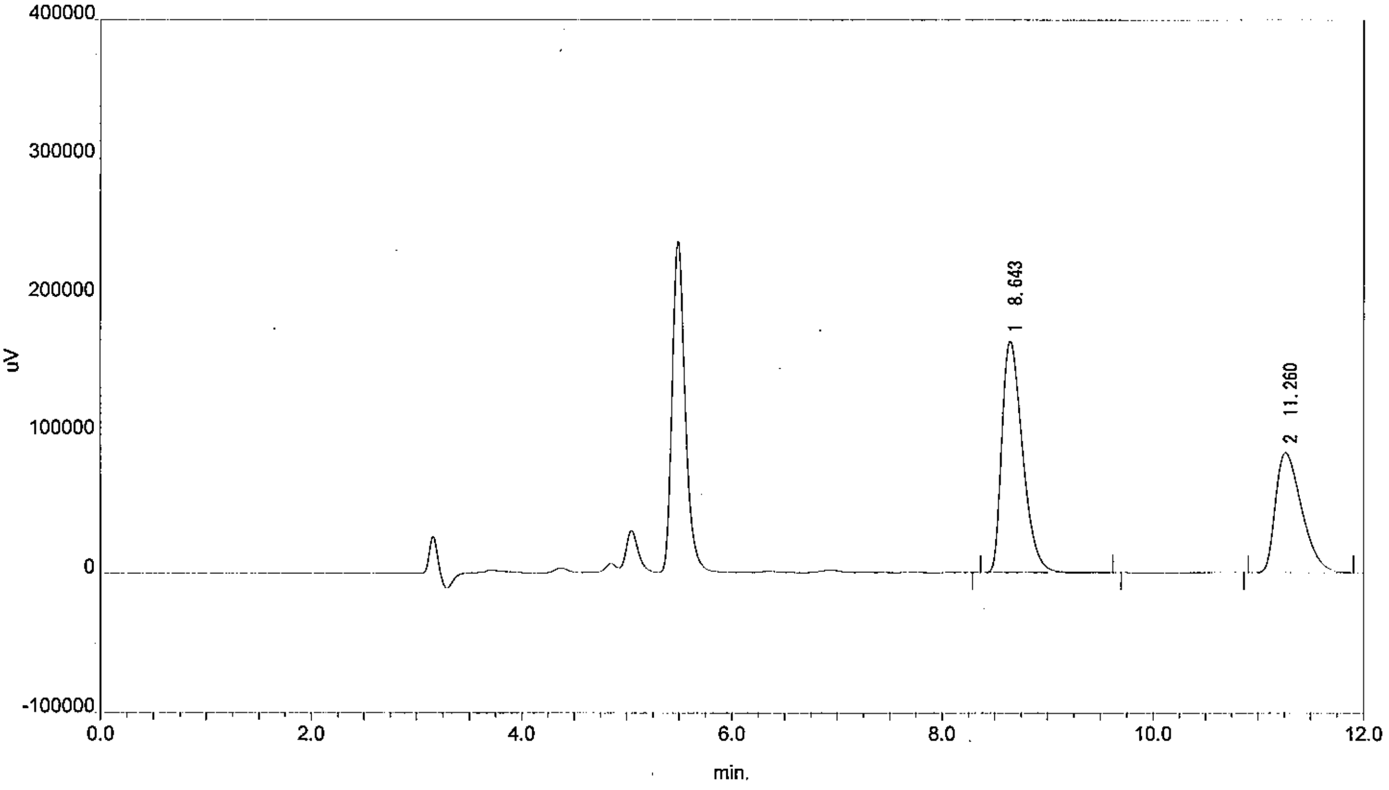

解析結果

No.	Rt(min)	ピーク名	面積	面積(%)	高さ	NTP	対称性	分離度
1	8.64		2266402.700	60.2957	166707	9093.8	1.464	6.343
2	11.26		1492410.824	39.7043	86188	9485.0	1.517	――――
			3758813.524	100.0000	252895			

Chromatogram Report

Table 2, Entry 1

分析条件

データファイル名	: 2020 0109 No.7 cl-rac (+)ADPE dioxane 再々測定取り直し2020Y01M09D11h07m36s.crm
解析ファイル名	:
チャンネルNo.	: 1
分析時間	: 19.9 min
取込間隔	: 200 msec
データ保存場所	: c:¥documents and settings¥user¥my documents¥クロマトプロデータ¥村松¥
分析日時	: Thu Jan 09 11:07:36 2020
コメント	: 2020 0109 No.7 cl-rac (+)ADPE dioxane

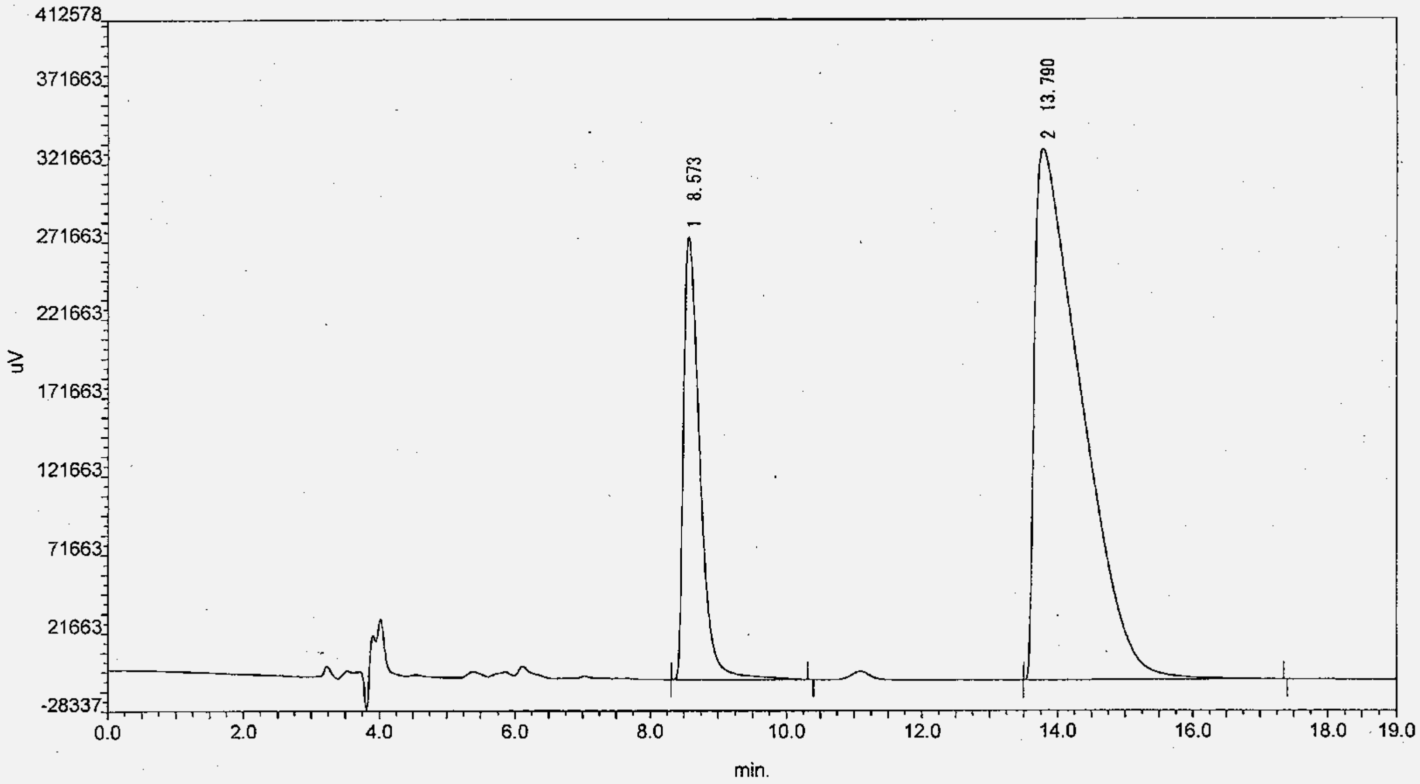

解析結果

No.	Rt(min)	ピーク名	面積	面積(%)	高さ	NTP	対称性	分離度
1	8.57		4699193.115	22.5359	282870	6290.9	1.847	6.028
2	13.79		16152839.241	77.4641	339171	1804.5	3.703	-----
			20852032.356	100.0000	622041			

Chromatogram Report

Table 2, Entry 2

分析条件

データファイル名	:	rac-2; (+)-ADPE; CHCl3-12022Y11M07D20h15m03s.crm
解析ファイル名	:	
チャンネルNo.	:	1
分析時間	:	20.0 min
取込間隔	:	200 msec
データ保存場所	:	c:¥users¥有機工業¥documents¥クロマトプロデータ¥srinivas¥second paper¥
分析日時	:	Mon Nov 07 20:15:03 2022
コメント	:	rac-2; (+)-ADPE; CHCl3-1

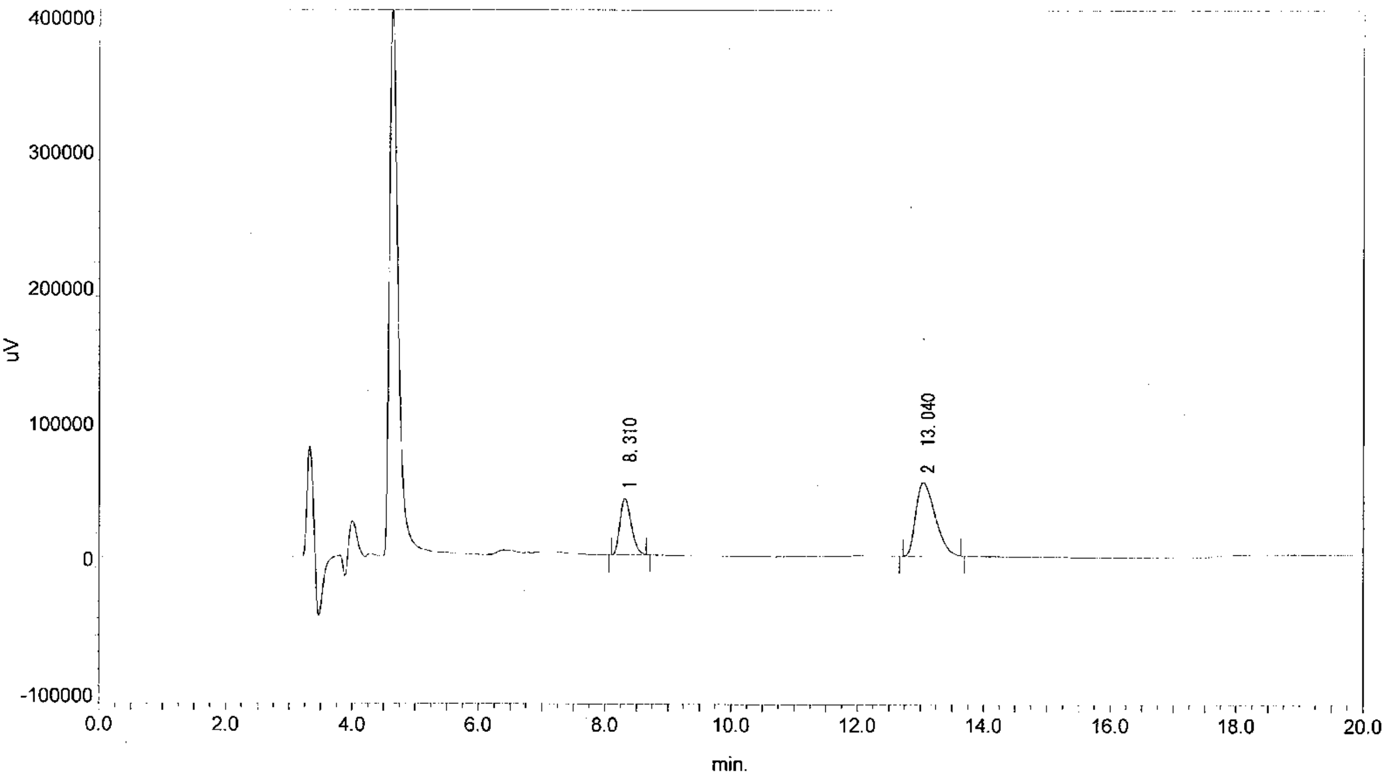

解析結果

No.	Rt(min)	ピーク名	面積	面積(%)	高さ	NTP	対称性	分離度
1	8.31		523810.632	31.0665	41870	9702.9	1.323	10.382
2	13.04		1162285.300	68.9335	54343	8266.2	1.469	――――
			1686095.932	100.0000	96213			

Chromatogram Report

Table 2, Entry 3

分析条件

データファイル名	:	rac-2; (+)-ADPE; AcOEt2022Y11M08D00h04m56s.crm
解析ファイル名	:	
チャンネルNo.	:	1
分析時間	:	20.0 min
取込間隔	:	200 msec
データ保存場所	:	c:¥users¥有機工業¥documents¥クロマトプロデータ¥srinivas¥second paper¥
分析日時	:	Tue Nov 08 00:04:56 2022
コメント	:	rac-2; (+)-ADPE; AcOEt

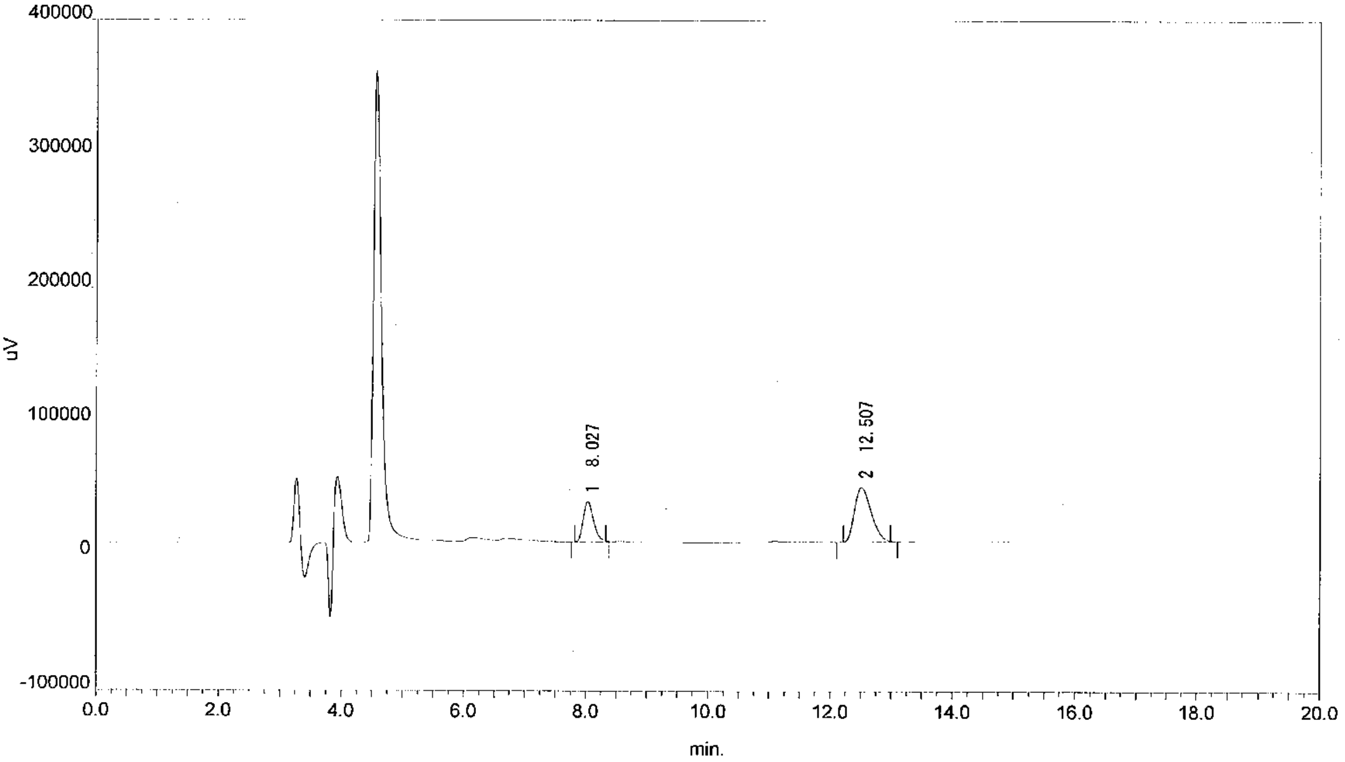

解析結果

No.	Rt(min)	ピーク名	面積	面積(%)	高さ	NTP	対称性	分離度
1	8.03		351567.287	31.1785	29915	10154.4	1.314	10.780
2	12.51		776027.146	68.8215	40687	9525.2	1.363	------
			1127594.433	100.0000	70602			

Chromatogram Report

Table 2, Entry 4

分析条件

データファイル名	: cl-カルボン酸メチル　(+)ADPE THF 精製後2019Y11M15D20h55m45s.crm
解析ファイル名	:
チャンネルNo.	: 1
分析時間	: 15.5 min
取込間隔	: 200 msec
データ保存場所	: c:¥documents and settings¥user¥my documents¥クロマトプロデータ¥村松¥
分析日時	: Fri Nov 15 20:55:45 2019
コメント	: cl-カルボン酸メチル　(+)ADPE THF 精製後

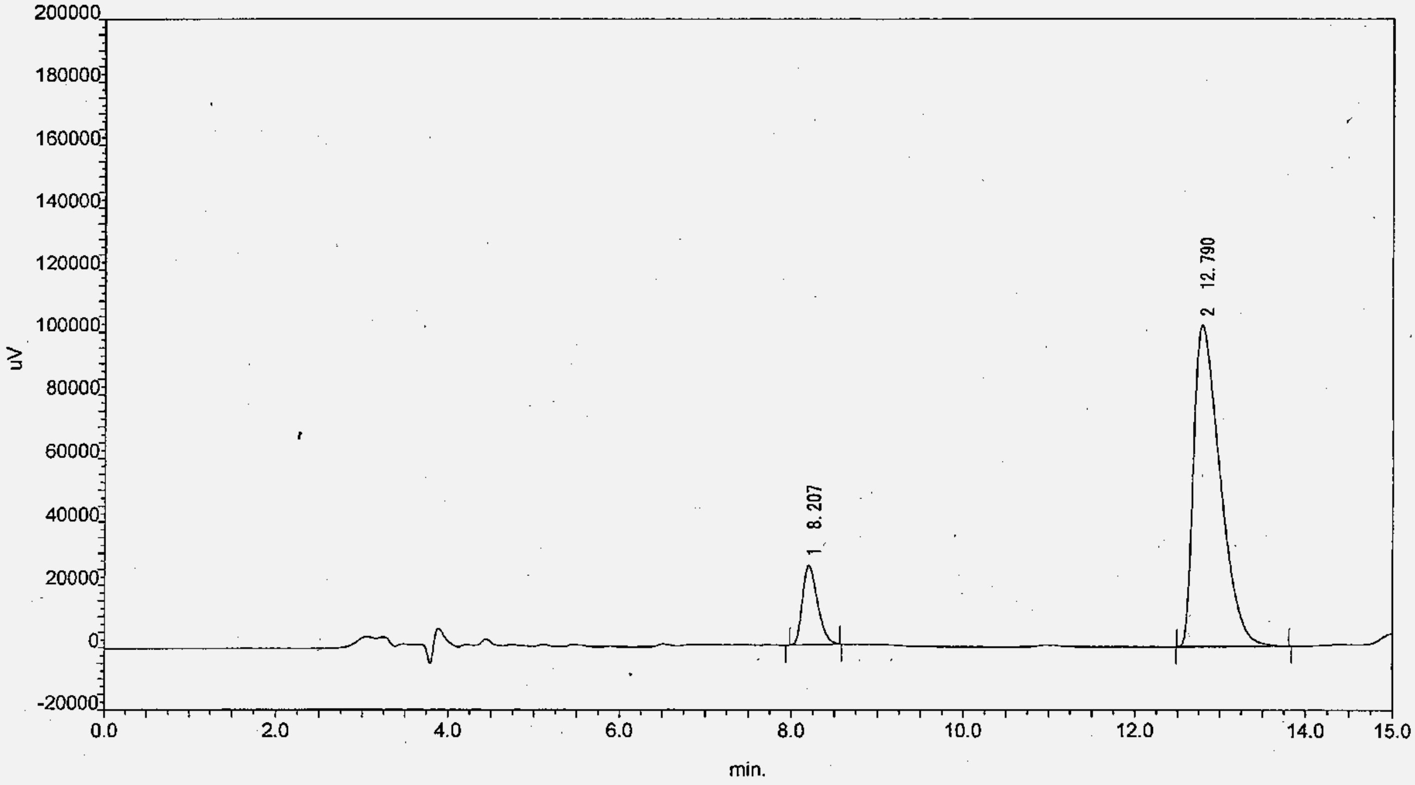

解析結果

No.	Rt(min)	ピーク名	面積	面積(%)	高さ	NTP	対称性	分離度
1	8.21		306428.536	12.1937	25154	9893.2	1.312	10.034
2	12.79		2206584.048	87.8063	101899	7685.6	1.790	――――
			2513012.584	100.0000	127053			

Chromatogram Report

Table 2, Entry 5

分析条件
データファイル名　: cl-カルボン酸メチル (+)ADPE IPA 取り直し2019Y11M25D16h33m16s.crm
解析ファイル名　:
チャンネルNo.　: 1
分析時間　: 15.0 min
取込間隔　: 200 msec
データ保存場所　: c:¥documents and settings¥user¥my documents¥クロマトプロデータ¥村松¥
分析日時　: Mon Nov 25 16:33:16 2019
コメント　: cl-カルボン酸メチル (+)ADPE IPA

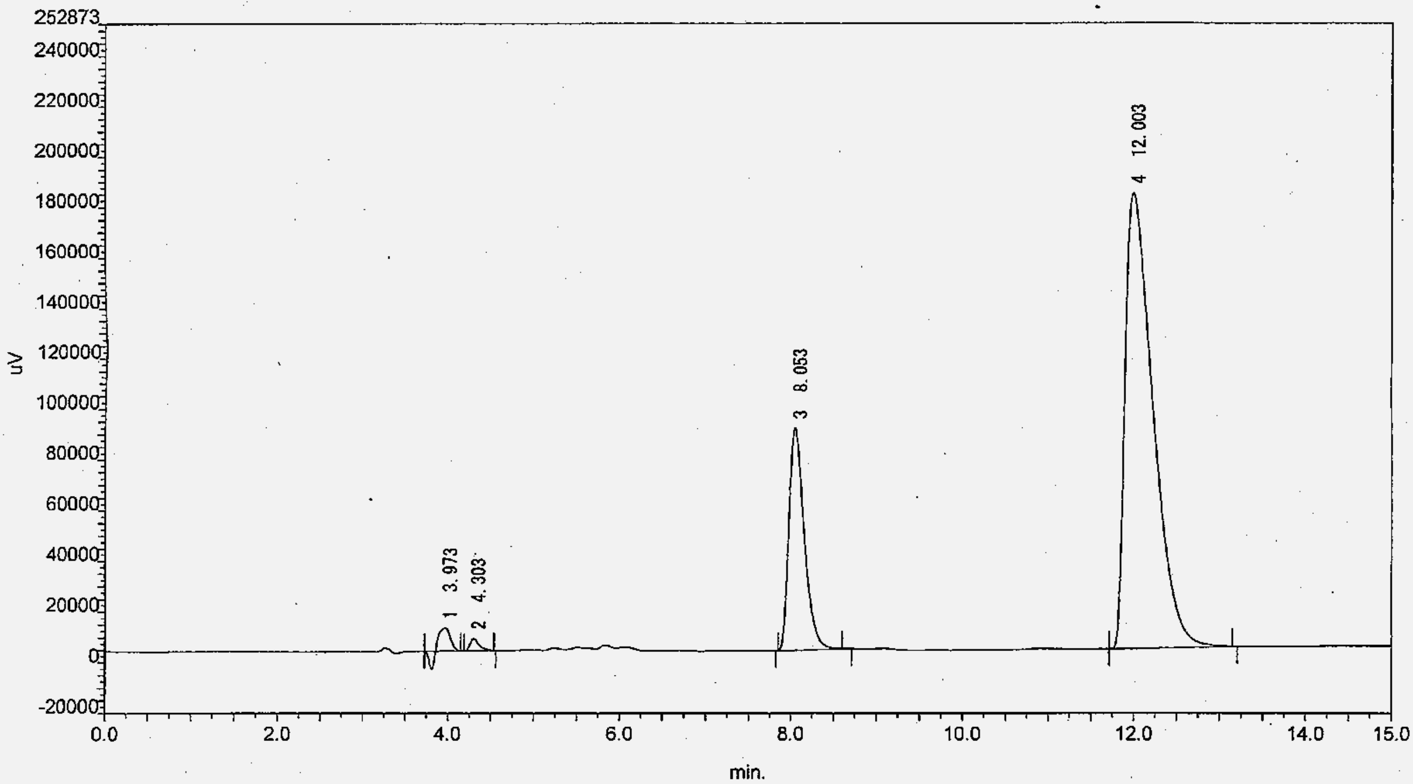

解析結果

No.	Rt(min)	ピーク名	面積	面積(%)	高さ	NTP	対称性	分離度
1	3.97		58887.883	1.1185	9145	8612.1	1.137	1.785
2	4.30		35492.768	0.6742	4756	7521.4	1.687	14.089
3	8.05		1099790.379	20.8898	87612	8888.4	1.446	8.317
4	12.00		4070547.114	77.3175	180147	6232.0	2.017	------
			5264718.144	100.0000	281660			

Chromatogram Report

Table 2, Entry 6

分析条件

データファイル名	: No.11 Cl-rac (+)ADPE H202020Y01M21D15h33m28s.crm
解析ファイル名	:
チャンネルNo.	: 1
分析時間	: 18.7 min
取込間隔	: 200 msec
データ保存場所	: c:¥documents and settings¥user¥my documents¥クロマトプロデータ¥村松¥
分析日時	: Tue Jan 21 15:33:28 2020
コメント	: No.11 Cl-rac (+)ADPE H20

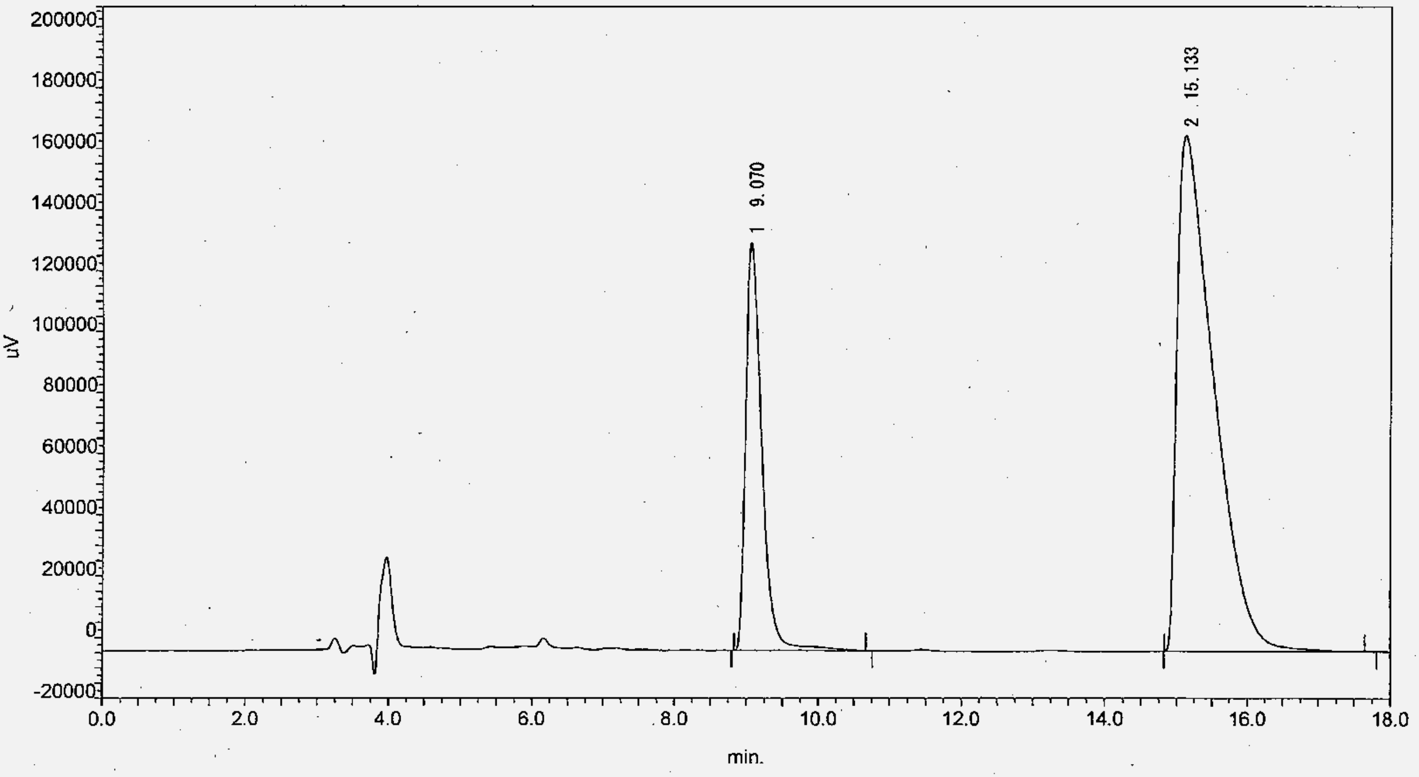

解析結果

No.	Rt(min)	ピーク名	面積	面積(%)	高さ	NTP	対称性	分離度
1	9.07		2087461.414	25.6318	133471	7814.2	1.624	8.723
2	15.13		6056583.476	74.3682	168585	3817.3	2.681	------
			8144044.890	100.0000	302056			

Chromatogram Report

Table 3, Entry 1

分析条件
データファイル名 : 2021.1.29 化合物1 (-)-ADPE dioxane2021Y01M27D12h23m44s.crm2021Y01M29D22h25m49:
解析ファイル名 :
チャンネルNo. : 1
分析時間 : 20.0 min
取込間隔 : 200 msec
データ保存場所 : c:¥documents and settings¥user¥my documents¥クロマトプロデータ¥kanda yusuke¥
分析日時 : Fri Jan 29 22:25:49 2021
コメント : [redacted]

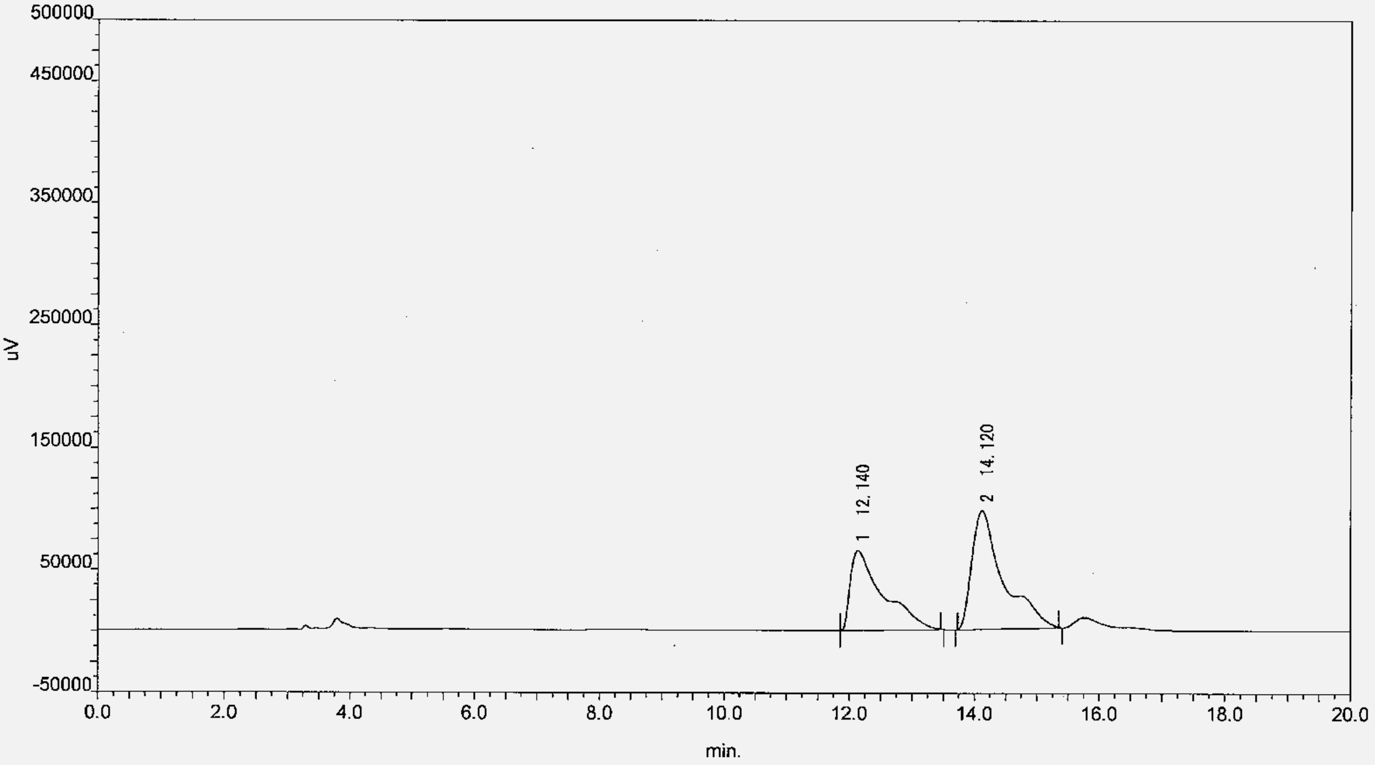

解析結果

No.	Rt(min)	ピーク名	面積	面積(%)	高さ	NTP	対称性	分離度
1	12.14		2330769.057	40.8927	65015	1816.6	3.040	1.721
2	14.12		3368948.784	59.1073	97254	2363.9	2.138	------
			5699717.841	100.0000	162269			

Chromatogram Report

Table 3, Entry 2

分析条件

データファイル名	: rac-3; (-)-ADPE; toluene-32022Y11M08D07h25m42s.crm
解析ファイル名	:
チャンネルNo.	: 1
分析時間	: 20.0 min
取込間隔	: 200 msec
データ保存場所	: c:¥users¥有機工業¥documents¥クロマトプロデータ¥srinivas¥second paper¥
分析日時	: Tue Nov 08 07:25:42 2022
コメント	: rac-3; (-)-ADPE; toluene-3

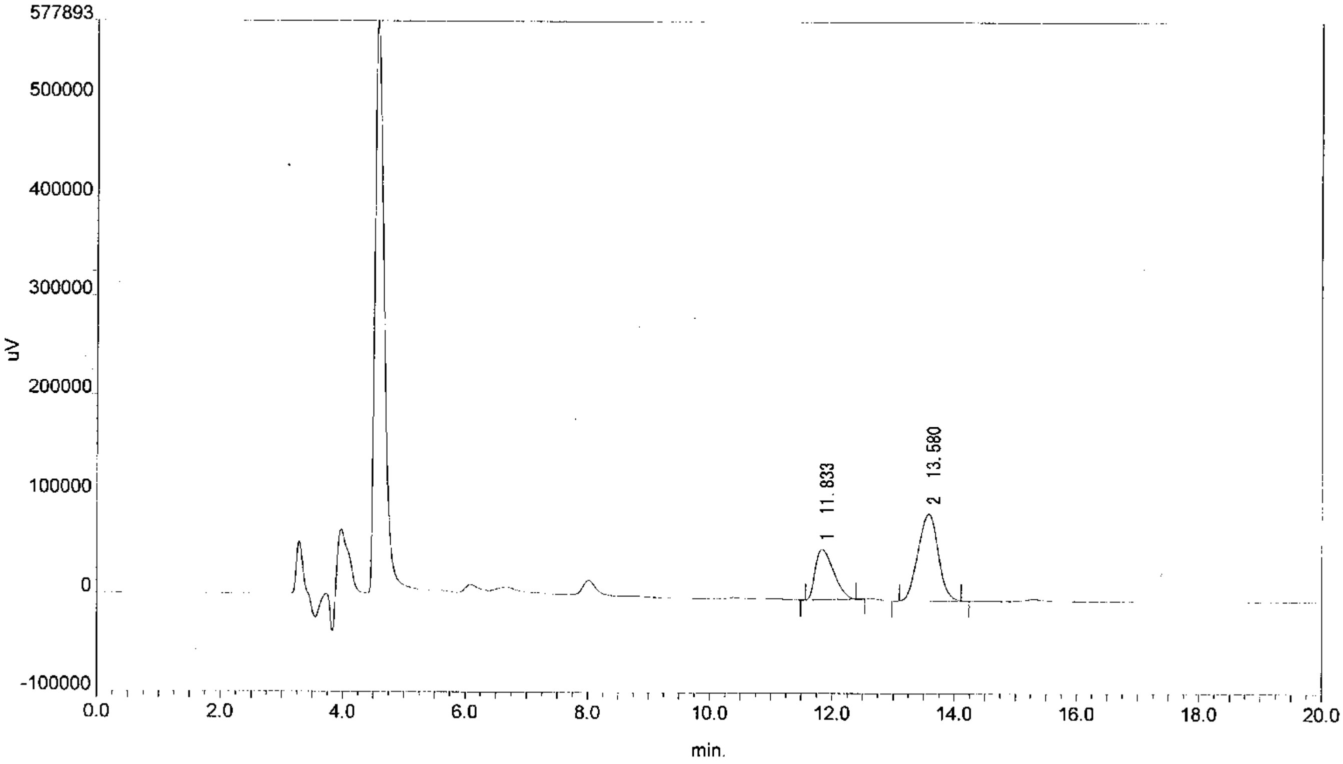

解析結果

No.	Rt(min)	ピーク名	面積	面積(%)	高さ	NTP	対称性	分離度
1	11.83		1046061.339	34.1292	50732	7447.9	1.571	3.020
2	13.58		2018943.486	65.8708	87894	7975.9	1.005	------
			3065004.825	100.0000	138626			

Chromatogram Report

Table 3, Entry 3

分析条件

データファイル名	:	2021.1.27 化合物1 (-)-ADPE CHCl32021Y01M27D12h23m44s.crm
解析ファイル名	:	
チャンネルNo.	:	1
分析時間	:	20.0 min
取込間隔	:	200 msec
データ保存場所	:	c:¥documents and settings¥user¥my documents¥クロマトプロデータ¥kanda yusuke¥
分析日時	:	Wed Jan 27 12:23:44 2021
コメント	:	2021.1.27 化合物1 (-)-ADPE ~~THF~~ $CHCl_3$ Ec 4.8/

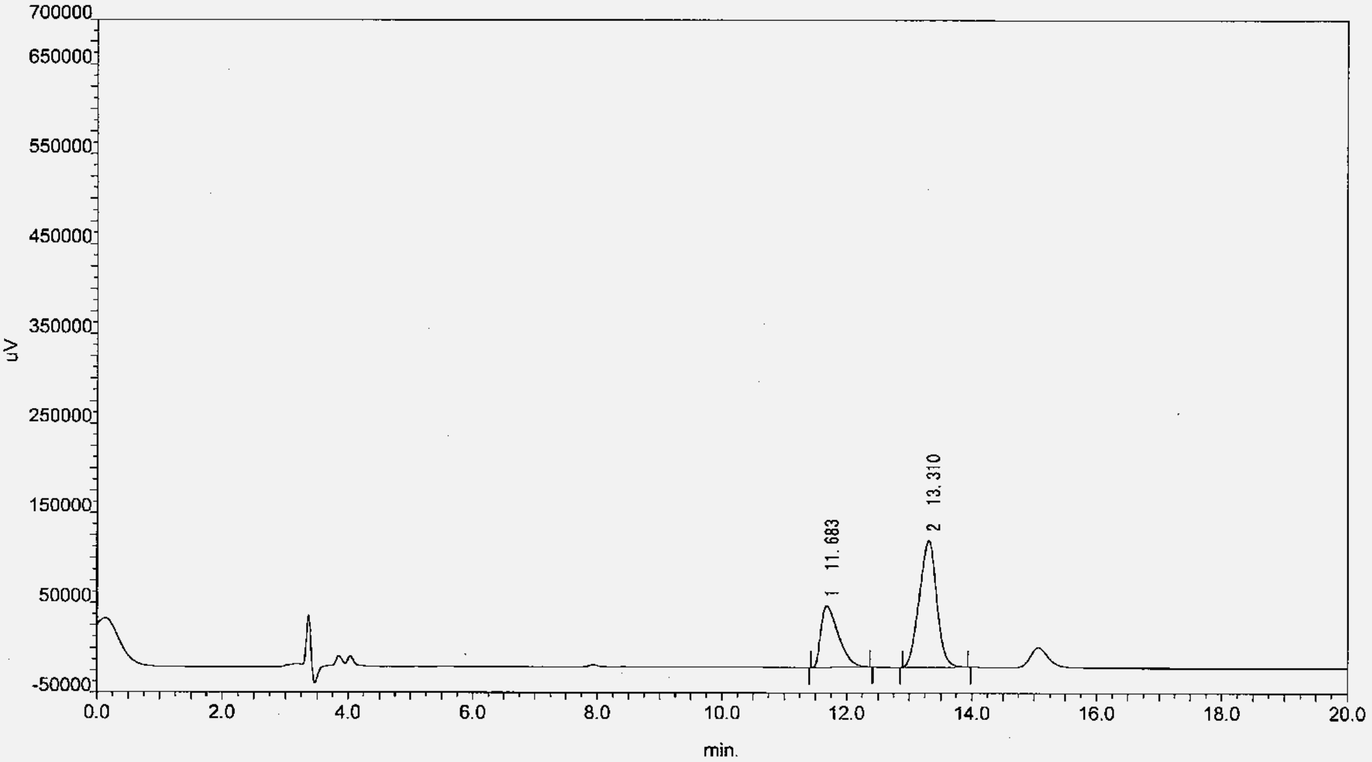

解析結果

No.	Rt(min)	ピーク名	面積	面積(%)	高さ	NTP	対称性	分離度
1	11.68		1344864.255	32.9340	68363	7655.6	1.814	3.069
2	13.31		2738652.991	67.0660	140817	10242.2	0.991	------
			4083517.246	100.0000	209180			

Chromatogram Report

Table 3, Entry 4

分析条件

データファイル名	:	2021.1.29 化合物1 (-)-ADPE AcOEt2021Y01M27D12h23m44s.crm2021Y01M29D22h05m33s.(
解析ファイル名	:	
チャンネルNo.	:	1
分析時間	:	20.0 min
取込間隔	:	200 msec
データ保存場所	:	c:¥documents and settings¥user¥my documents¥クロマトプロデータ¥kanda yusuke¥
分析日時	:	Fri Jan 29 22:05:33 2021
コメント	:	[redacted]

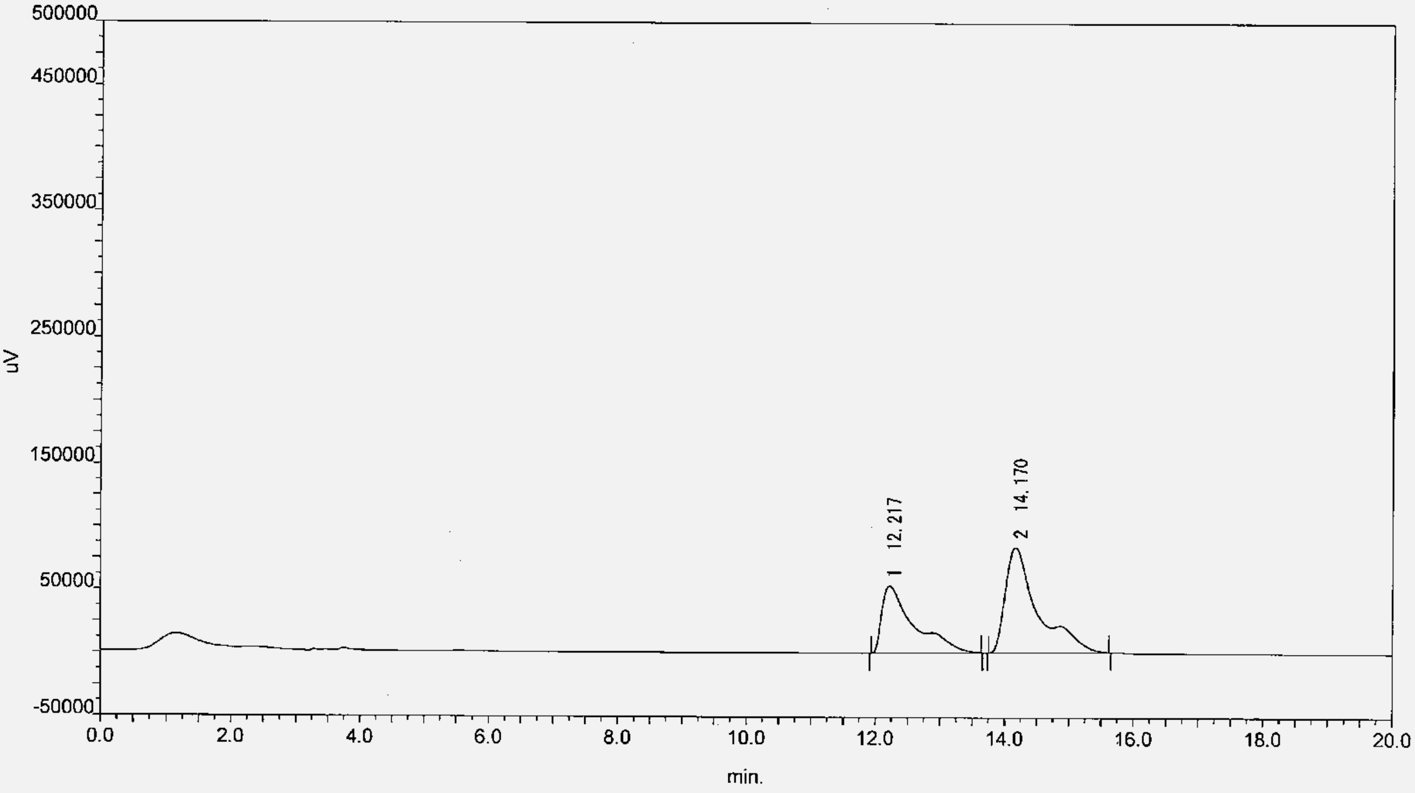

解析結果

No.	Rt(min)	ピーク名	面積	面積(%)	高さ	NTP	対称性	分離度
1	12.22		1839275.916	38.6857	52879	1797.9	3.096	1.653
2	14.17		2915126.557	61.3143	83438	2188.7	2.284	------
			4754402.473	100.0000	136317			

Chromatogram Report

Table 3, Entry 5

分析条件
データファイル名 : 2021.1.27 化合物1 (−)-ADPE THF2021Y01M27D12h02m22s.crm
解析ファイル名 :
チャンネルNo. : 1
分析時間 : 20.0 min
取込間隔 : 200 msec
データ保存場所 : c:¥documents and settings¥user¥my documents¥クロマトプロデータ¥kanda yusuke¥
分析日時 : Wed Jan 27 12:02:22 2021
コメント : 2021.1.27 化合物1 (−)-ADPE THF Ec 7.6

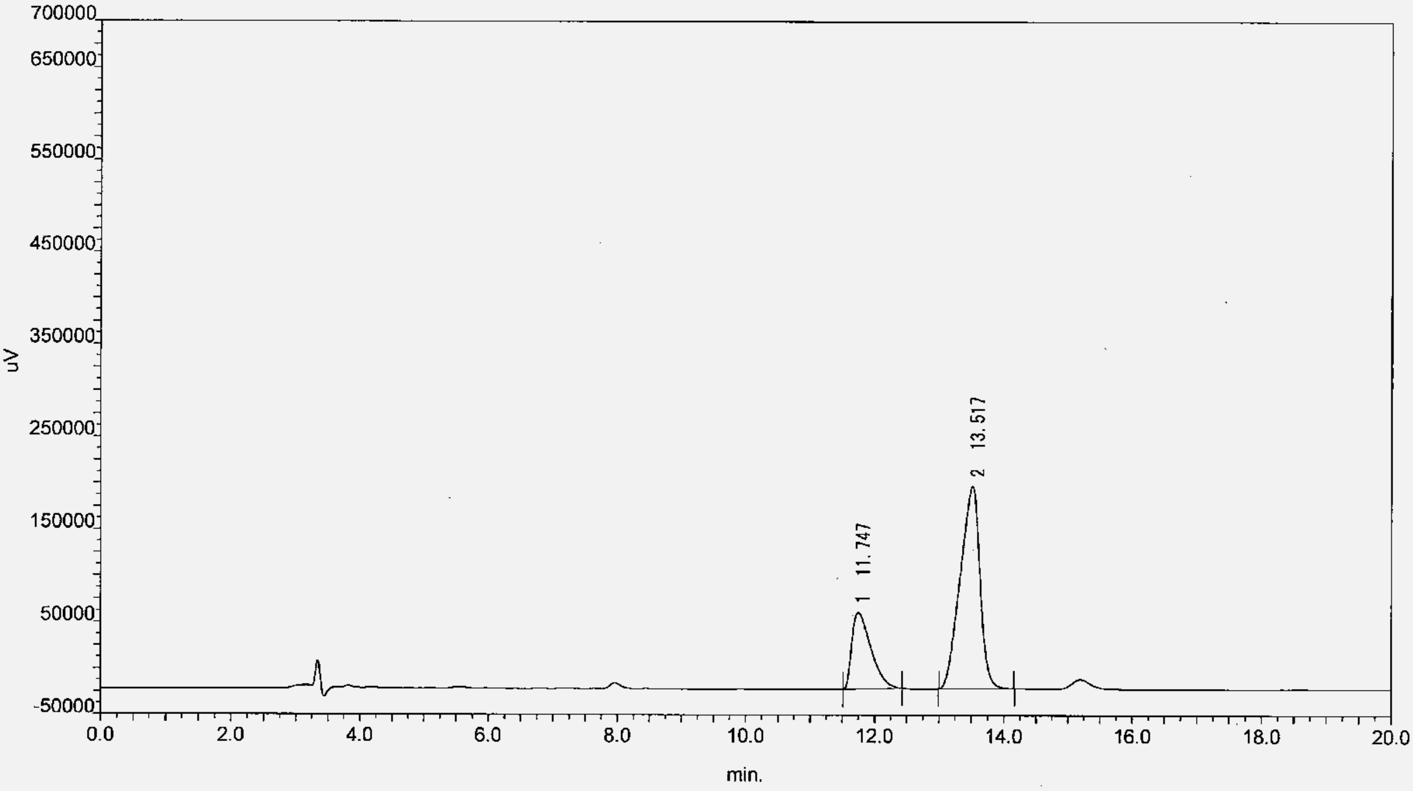

解析結果

No.	Rt(min)	ピーク名	面積	面積(%)	高さ	NTP	対称性	分離度
1	11.75		1683841.400	26.4989	82526	7209.8	1.871	3.131
2	13.52		4670551.827	73.5011	218742	8776.2	0.849	------
			6354393.227	100.0000	301268			

Chromatogram Report

Table 3, Entry 6

分析条件

データファイル名	:	2021.1.29 化合物1 (-)-ADPE iPrOH2021Y01M27D12h23m44s.crm2021Y01M29D21h43m49s.[illegible]
解析ファイル名	:	
チャンネルNo.	:	1
分析時間	:	20.0 min
取込間隔	:	200 msec
データ保存場所	:	c:¥documents and settings¥user¥my documents¥クロマトプロデータ¥kanda yusuke¥
分析日時	:	Fri Jan 29 21:43:49 2021
コメント	:	2021.1.29 化合物1 (-)-ADPE iPrOH

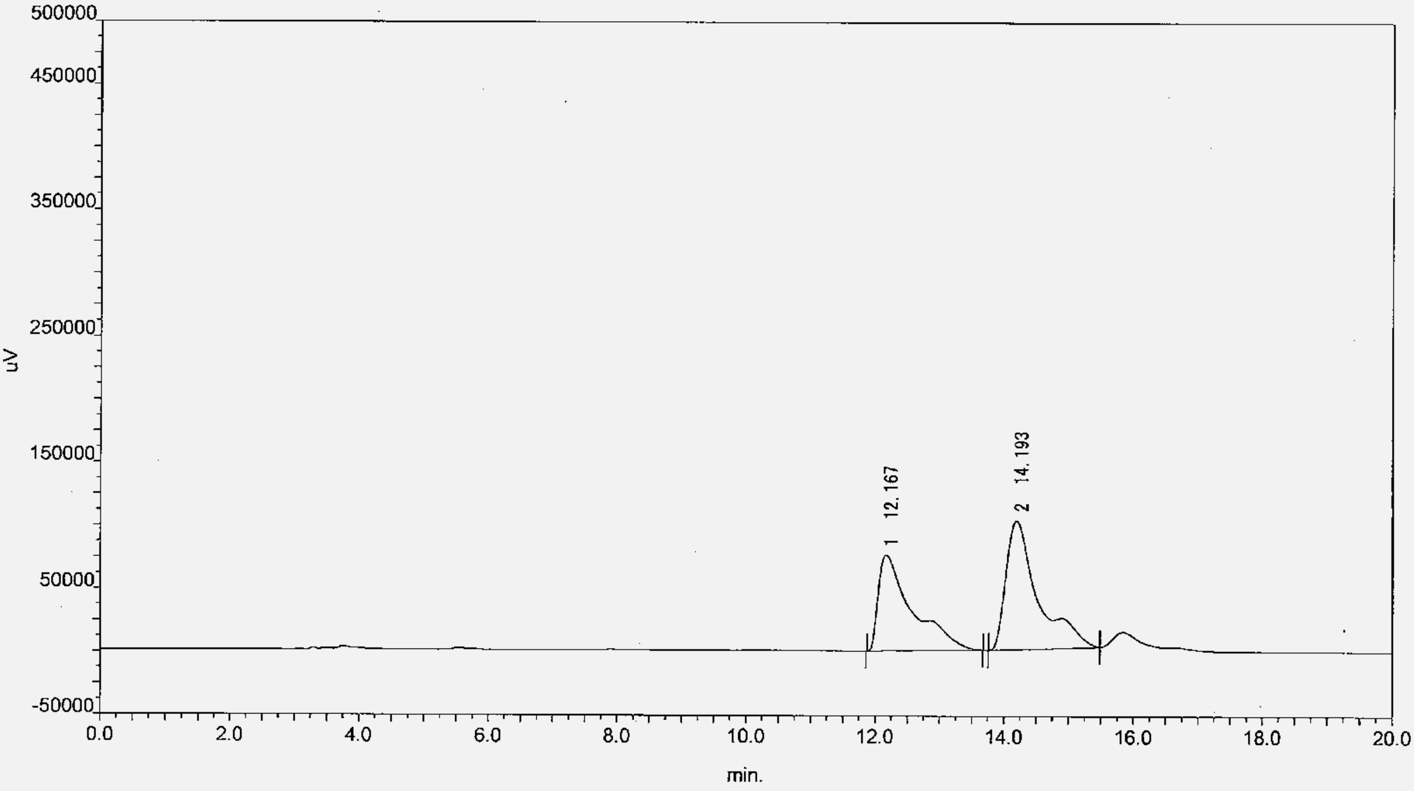

解析結果

No.	Rt(min)	ピーク名	面積	面積(%)	高さ	NTP	対称性	分離度
1	12.17		2799655.082	44.2626	76072	1626.9	3.191	1.683
2	14.19		3525442.326	55.7374	102194	2233.6	2.150	―――
			6325097.408	100.0000	178266			

Chromatogram Report

Table 3, Entry 7

分析条件
データファイル名 : rac-3; (-)-ADPE; EtOH2022Y11M17D19h36m26s.crm
解析ファイル名 :
チャンネルNo. : 1
分析時間 : 20.0 min
取込間隔 : 200 msec
データ保存場所 : c:¥users¥有機工業¥documents¥クロマトプロデータ¥srinivas¥second paper¥
分析日時 : Thu Nov 17 19:36:26 2022
コメント : rac-3; (-)-ADPE; EtOH

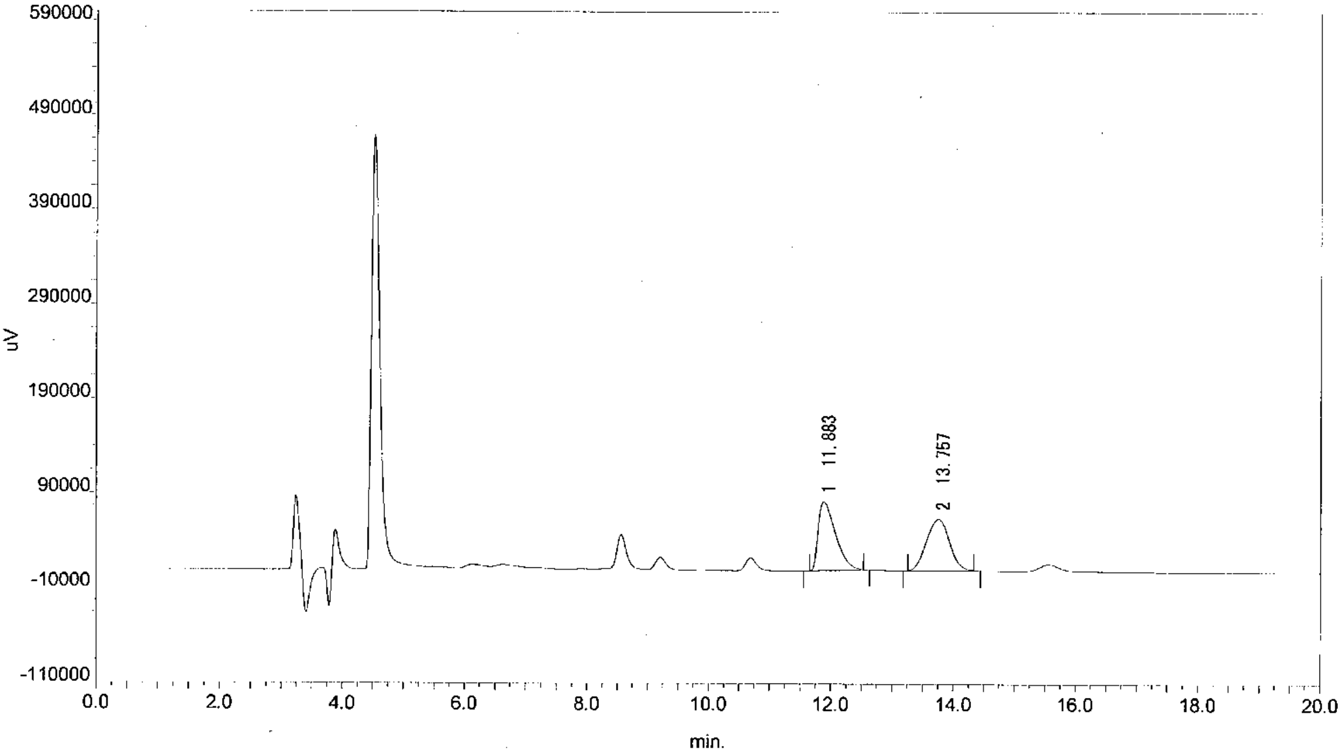

解析結果

No.	Rt(min)	ピーク名	面積	面積(%)	高さ	NTP	対称性	分離度
1	11.88		1427784.788	50.0265	72899	7994.1	1.934	3.065
2	13.76		1426274.495	49.9735	54363	6347.9	1.032	------
			2854059.282	100.0000	127262			

Chromatogram Report

Table 4, Entry 1

分析条件

データファイル名	: rac-1; cin; dioxane2022Y11M17D18h51m32s.crm
解析ファイル名	:
チャンネルNo.	: 1
分析時間	: 20.0 min
取込間隔	: 200 msec
データ保存場所	: c:¥users¥有機工業¥documents¥クロマトプロデータ¥srinivas¥second paper¥
分析日時	: Thu Nov 17 18:51:32 2022
コメント	: rac-1; cin; dioxane

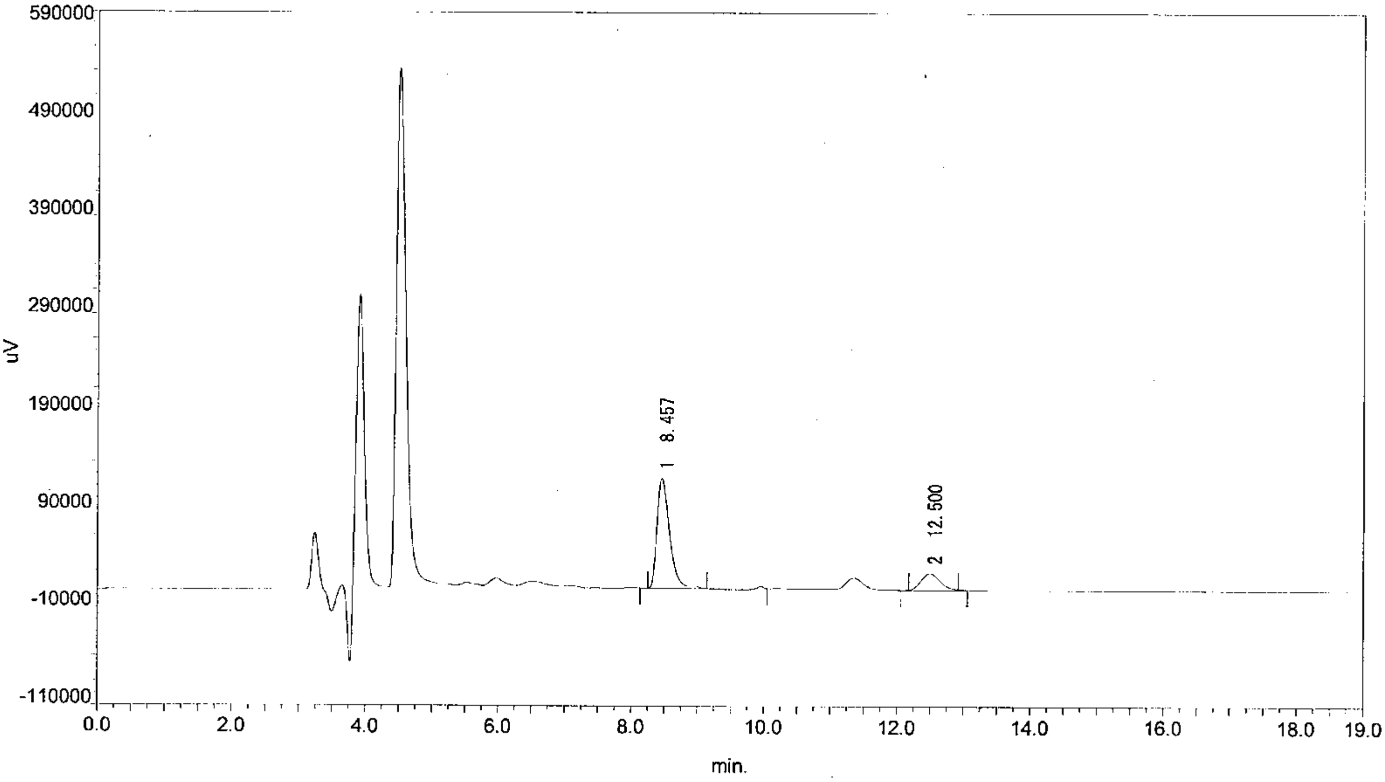

解析結果

No.	Rt(min)	ピーク名	面積	面積(%)	高さ	NTP	対称性	分離度
1	8.46		1475304.376	81.3577	112676	9372.9	1.553	9.175
2	12.50		338051.610	18.6423	17200	8833.7	1.196	―――
			1813355.986	100.0000	129876			

Chromatogram Report

Table 4, Entry 2

分析条件

データファイル名	:	シンコニジン(AcOEt)2016Y11M14D13h27m02s.crm
解析ファイル名	:	
チャンネルNo.	:	1
分析時間	:	17.4 min
取込間隔	:	200 msec
データ保存場所	:	c:¥users¥有機工業¥documents¥クロマトプロデータ¥yamazaki¥分割¥
分析日時	:	Mon Nov 14 13:27:02 2016
コメント	:	シンコニジン(AcOEt)

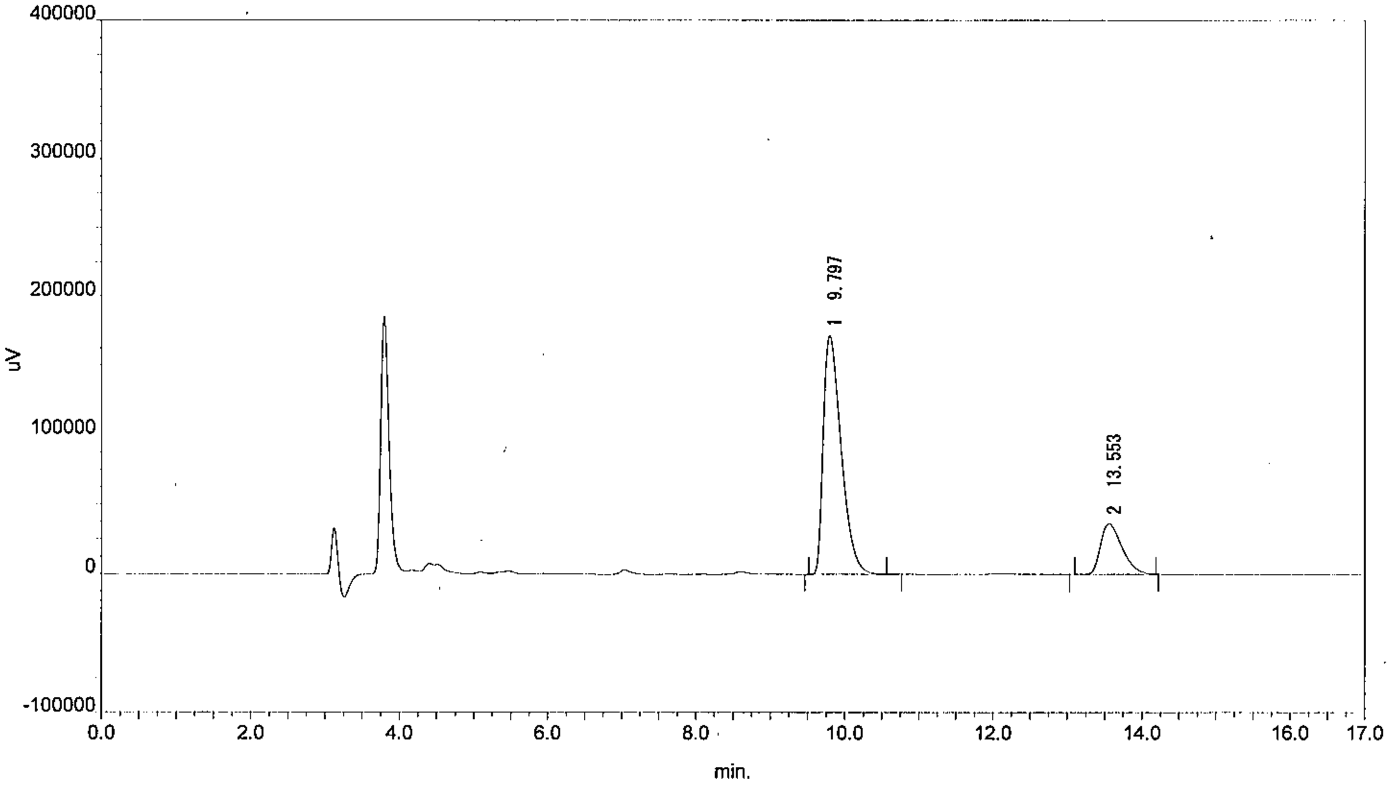

解析結果

No.	Rt(min)	ピーク名	面積	面積(%)	高さ	NTP	対称性	分離度
1	9.80		2861512.600	79.6595	171996	7691.0	1.644	7.611
2	13.55		730668.337	20.3405	36352	10066.7	1.470	------
			3592180.937	100.0000	208348			

Chromatogram Report

Table 4, Entry 3

分析条件

データファイル名	:	rac-1; cin; THF repeat 12022Y11M17D17h28m21s.crm
解析ファイル名	:	
チャンネルNo.	:	1
分析時間	:	20.0 min
取込間隔	:	200 msec
データ保存場所	:	c:¥users¥有機工業¥documents¥クロマトプロデータ¥srinivas¥second paper¥
分析日時	:	Thu Nov 17 17:28:21 2022
コメント	:	rac-2; cin; DIOXANE-11

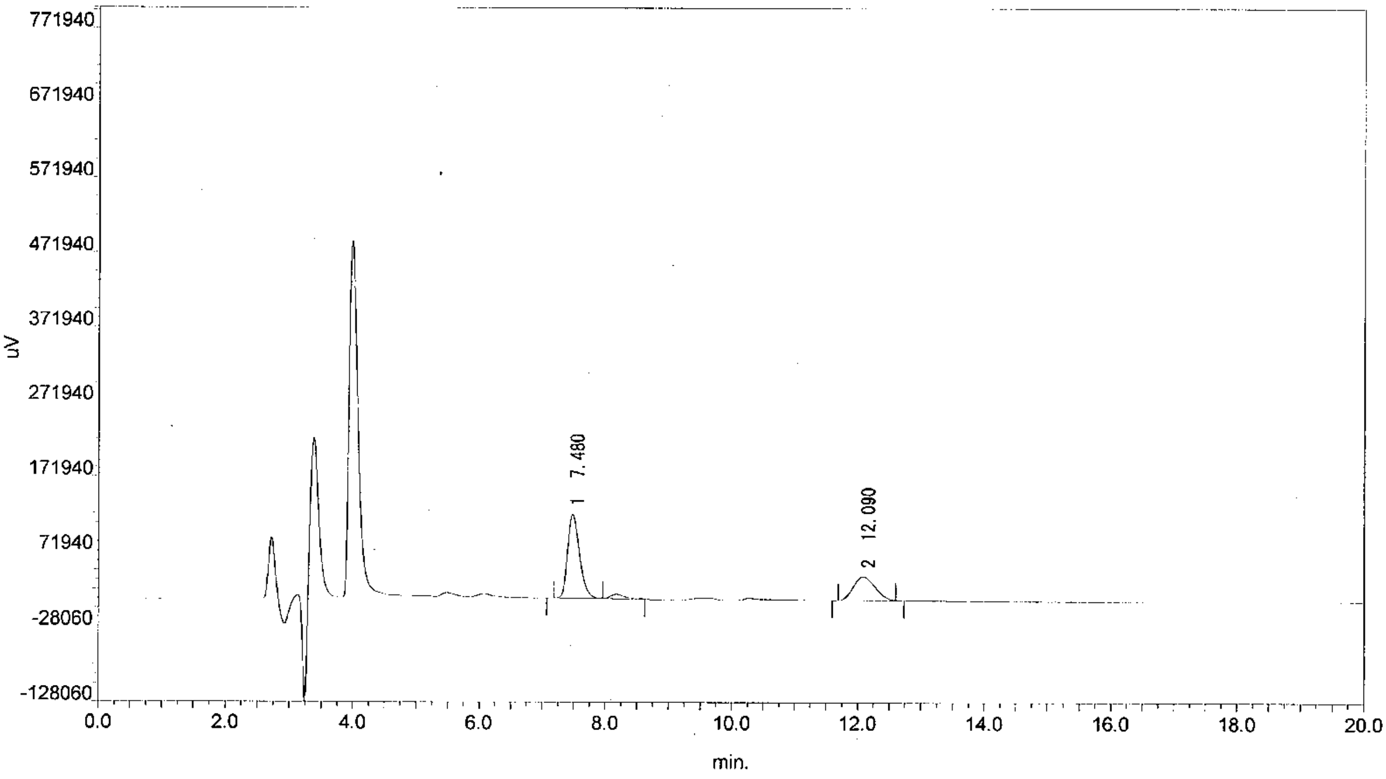

解析結果

No.	Rt(min)	ピーク名	面積	面積(%)	高さ	NTP	対称性	分離度
1	7.48		1483007.904	66.3367	112061	7149.0	1.347	9.297
2	12.09		752567.921	33.6633	31233	5747.3	1.168	------
			2235575.825	100.0000	143294			

Chromatogram Report

Table 4, Entry 4

分析条件

データファイル名	:	シンコニジン(2-PrOH)取り直し2016Y11M14D14h48m43s.crm
解析ファイル名	:	
チャンネルNo.	:	1
分析時間	:	15.0 min
取込間隔	:	200 msec
データ保存場所	:	c:¥users¥有機工業¥documents¥クロマトプロデータ¥yamazaki¥分割¥
分析日時	:	Mon Nov 14 14:48:43 2016
コメント	:	シンコニジン(2-PrOH)

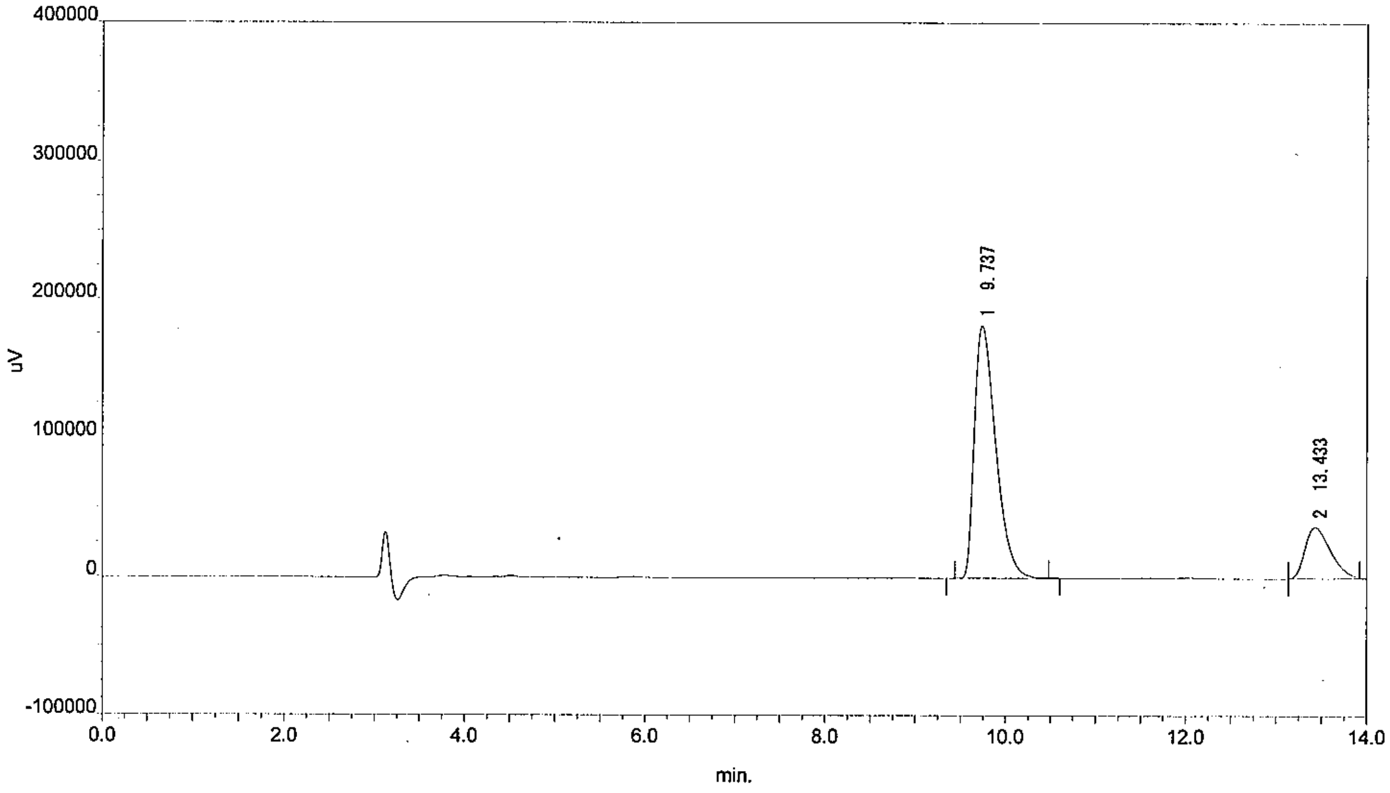

解析結果

No.	Rt(min)	ピーク名	面積	面積(%)	高さ	NTP	対称性	分離度
1	9.74		3015019.698	80.5944	181746	7654.1	1.653	7.594
2	13.43		725958.730	19.4056	37004	10340.3	1.436	------
			3740978.428	100.0000	218750			

Chromatogram Report

Table 4, Entry 5

分析条件

データファイル名	:	シンコニジン(EtOH)2016Y10M18D12h22m20s.crm
解析ファイル名	:	
チャンネルNo.	:	1
分析時間	:	17.3 min
取込間隔	:	200 msec
データ保存場所	:	c:¥users¥有機工業¥documents¥クロマトプロデータ¥yamazaki¥分割¥
分析日時	:	Tue Oct 18 12:22:20 2016
コメント	:	シンコニジン(EtOH)

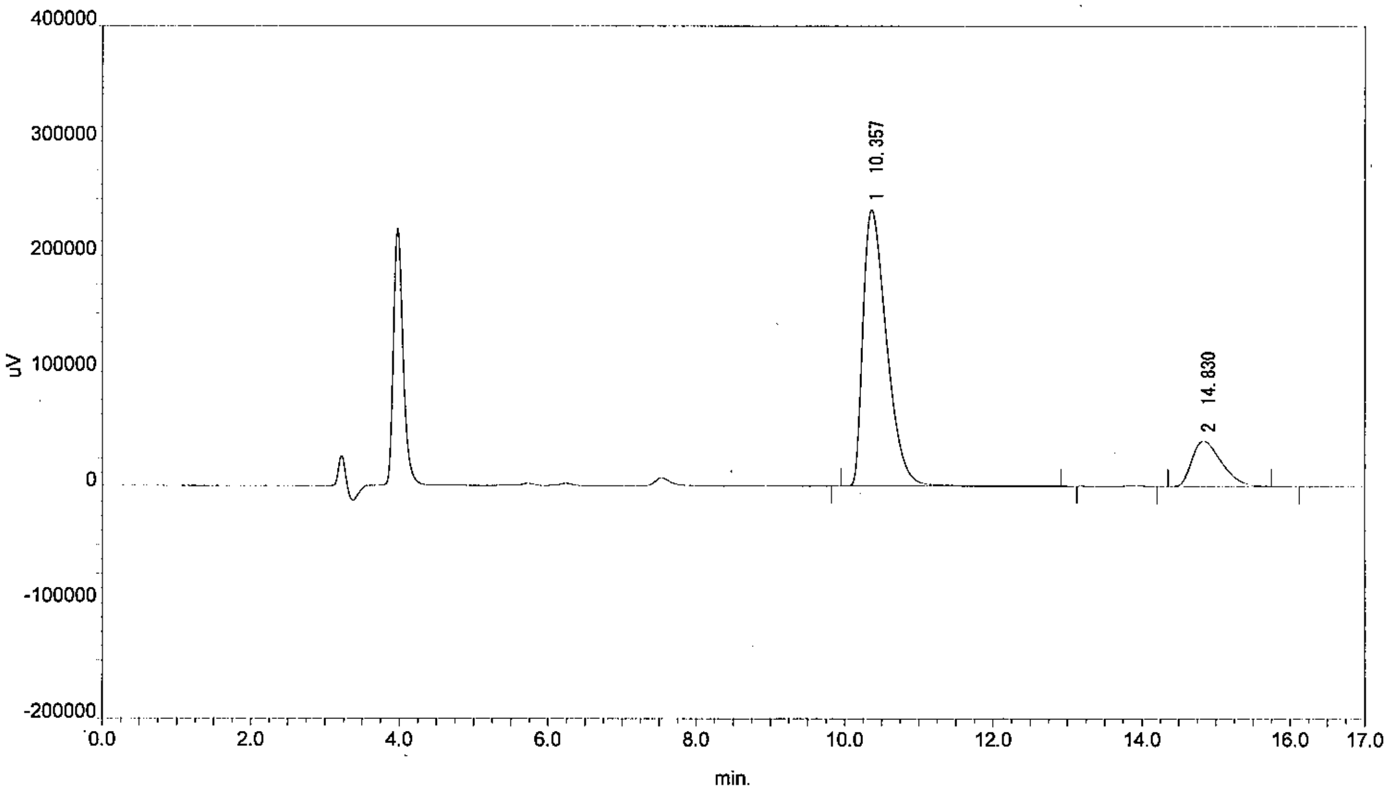

解析結果

No.	Rt(min)	ピーク名	面積	面積(%)	高さ	NTP	対称性	分離度
1	10.36		5309168.437	82.9542	239395	4926.2	1.652	6.680
2	14.83		1090948.948	17.0458	39109	6272.5	1.488	------
			6400117.385	100.0000	278504			

Chromatogram Report

Table 5, Entry 1

分析条件

データファイル名	: rac-2; cin; dioxane repeat 12022Y11M17D16h47m10s.crm
解析ファイル名	:
チャンネルNo.	: 1
分析時間	: 20.0 min
取込間隔	: 200 msec
データ保存場所	: c:¥users¥有機工業¥documents¥クロマトプロデータ¥srinivas¥second paper¥
分析日時	: Thu Nov 17 16:47:10 2022
コメント	: rac-1; cin; THF-1

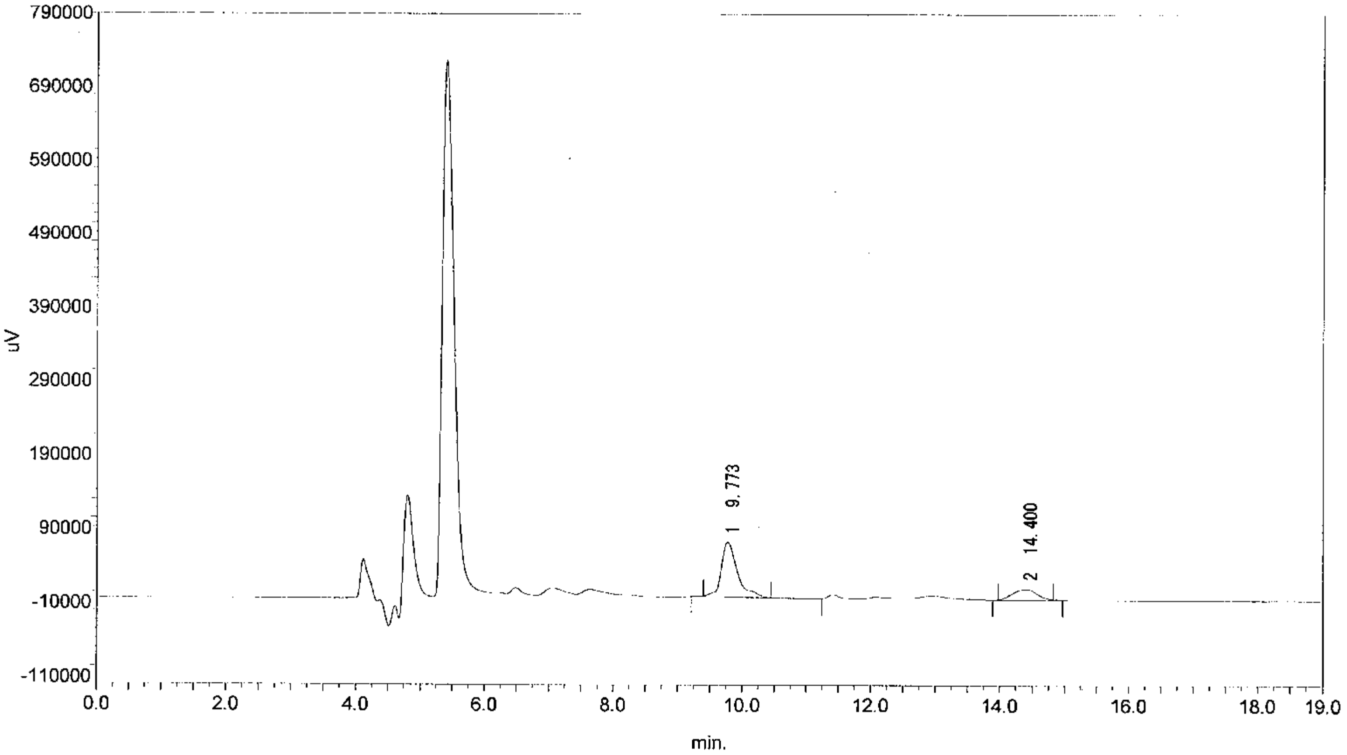

解析結果

No.	Rt(min)	ピーク名	面積	面積(%)	高さ	NTP	対称性	分離度
1	9.77		1292138.162	77.5316	74185	7018.7	1.377	8.155
2	14.40		374457.306	22.4684	14383	7432.6	1.030	------
			1666595.468	100.0000	88568			

Chromatogram Report

Table 5, Entry 2

分析条件

データファイル名	:	分割Cl-2,Cinchonidine,CHCl3,2019Y05M16D15h12m50s.crm
解析ファイル名	:	
チャンネルNo.	:	1
分析時間	:	17.7 min
取込間隔	:	200 msec
データ保存場所	:	c:¥users¥有機工業¥documents¥クロマトプロデータ¥卒業生¥丹保¥cl-ブタン酸分割¥
分析日時	:	Thu May 16 15:12:50 2019
コメント	:	分割Cl-2,Cinchonidine,CHCl3,

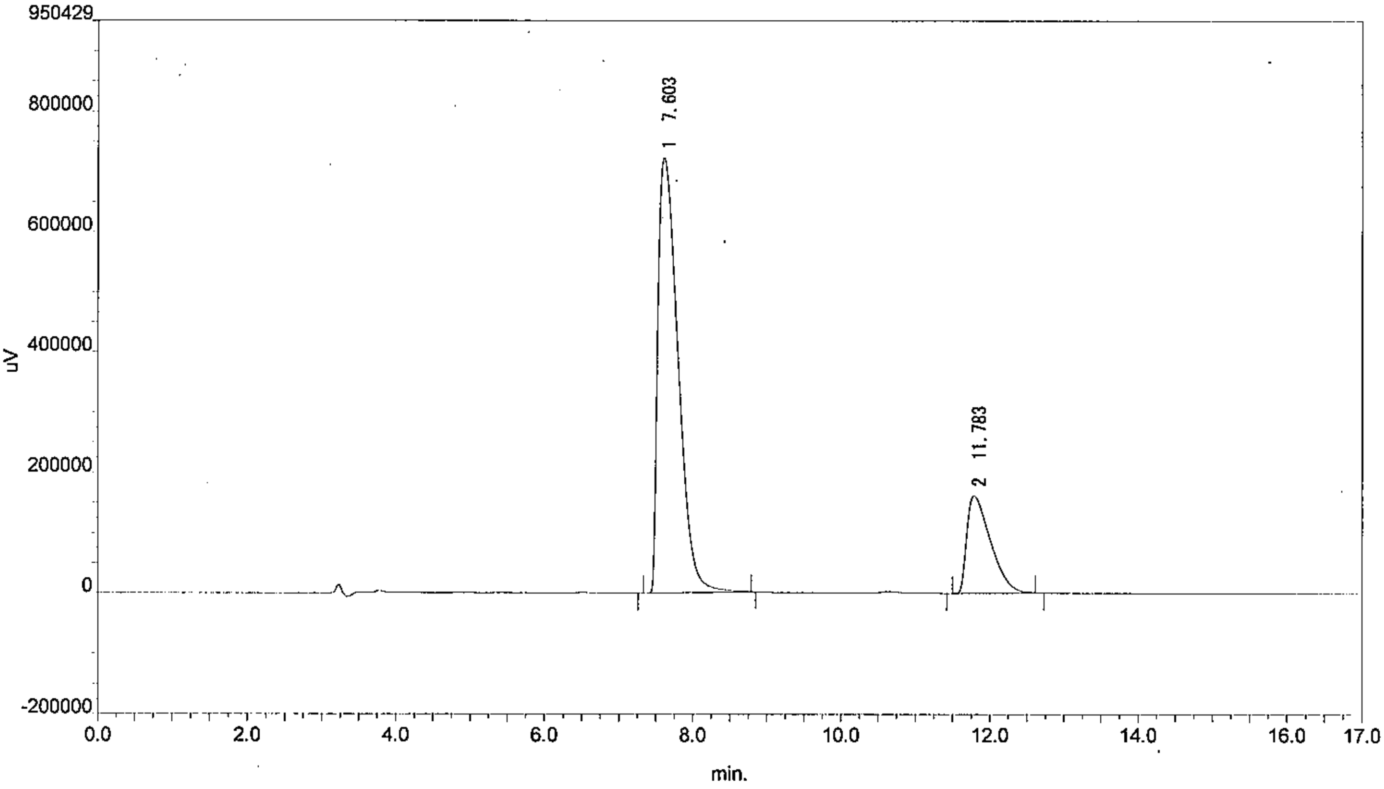

解析結果

No.	Rt(min)	ピーク名	面積	面積(%)	高さ	NTP	対称性	分離度
1	7.60		13771300.402	79.3149	723440	3940.7	2.110	7.727
2	11.78		3591505.079	20.6851	162343	6225.2	2.061	------
			17362805.481	100.0000	885783			

Chromatogram Report

Table 5, Entry 3

分析条件

データファイル名	:	OD-3 Cl-Ph AcoEtやり直し2017Y12M20D17h17m26s.crm
解析ファイル名	:	
チャンネルNo.	:	1
分析時間	:	30.0 min
取込間隔	:	200 msec
データ保存場所	:	c:¥documents and settings¥user¥my documents¥クロマトプロデータ¥yamazaki¥クロロ置
分析日時	:	Wed Dec 20 17:17:26 2017
コメント	:	OD-3 Cl-Ph AcoEtやり直し

Table 5-21, entry 1

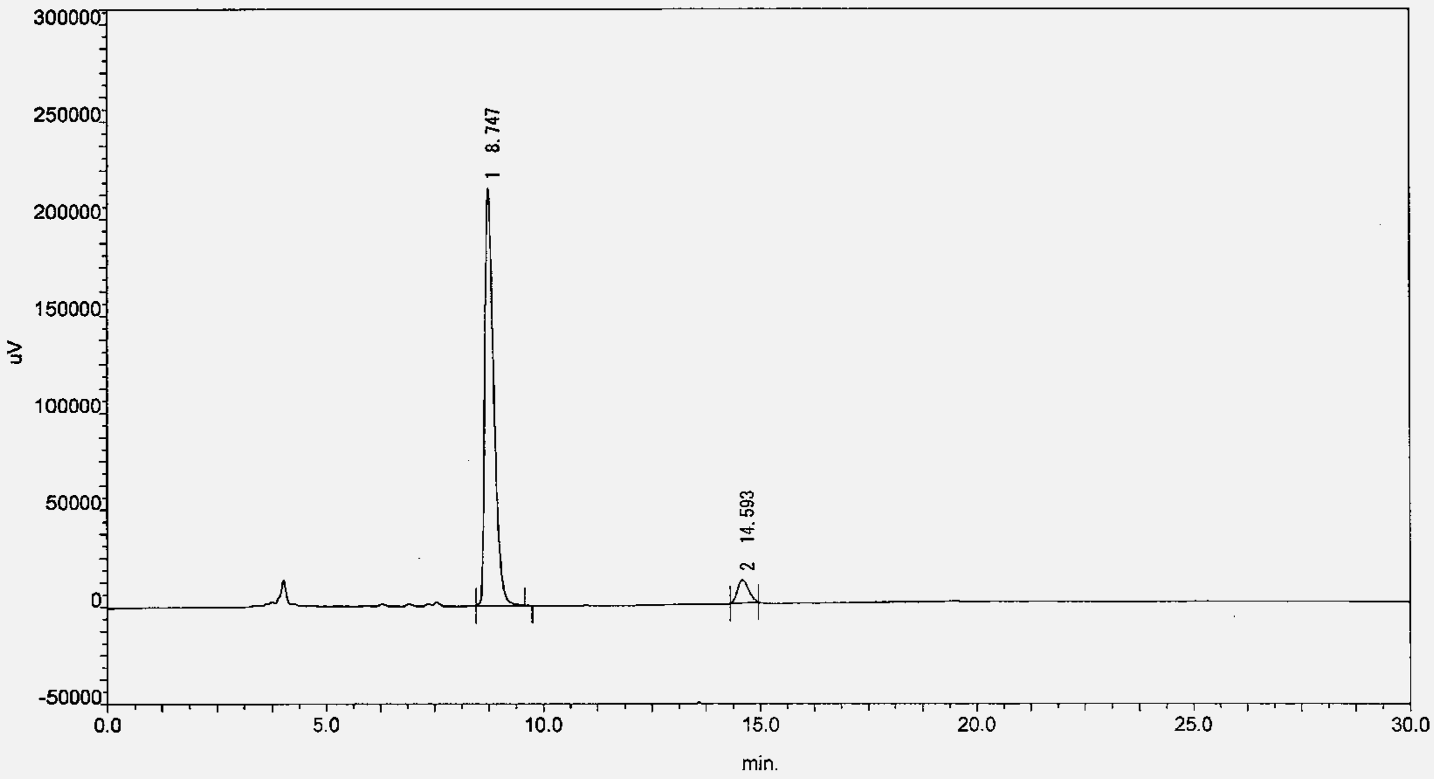

解析結果

No.	Rt(min)	ピーク名	面積	面積(%)	高さ	NTP	対称性	分離度
1	8.75		3109328.440	93.6506	215037	8131.7	1.589	13.458
2	14.59		210808.200	6.3494	11731	14735.0	1.187	-------
			3320136.640	100.0000	226768			

Chromatogram Report

Table 5, Entry 4

分析条件

データファイル名	:	分割Cl-2,Cinchonidine,THF,2019Y05M16D15h42m15s.crm
解析ファイル名	:	
チャンネルNo.	:	1
分析時間	:	15.5 min
取込間隔	:	200 msec
データ保存場所	:	c:¥users¥有機工業¥documents¥クロマトプロデータ¥卒業生¥丹保¥cl-ブタン酸分割¥
分析日時	:	Thu May 16 15:42:15 2019
コメント	:	分割Cl-2,Cinchonidine,THF,

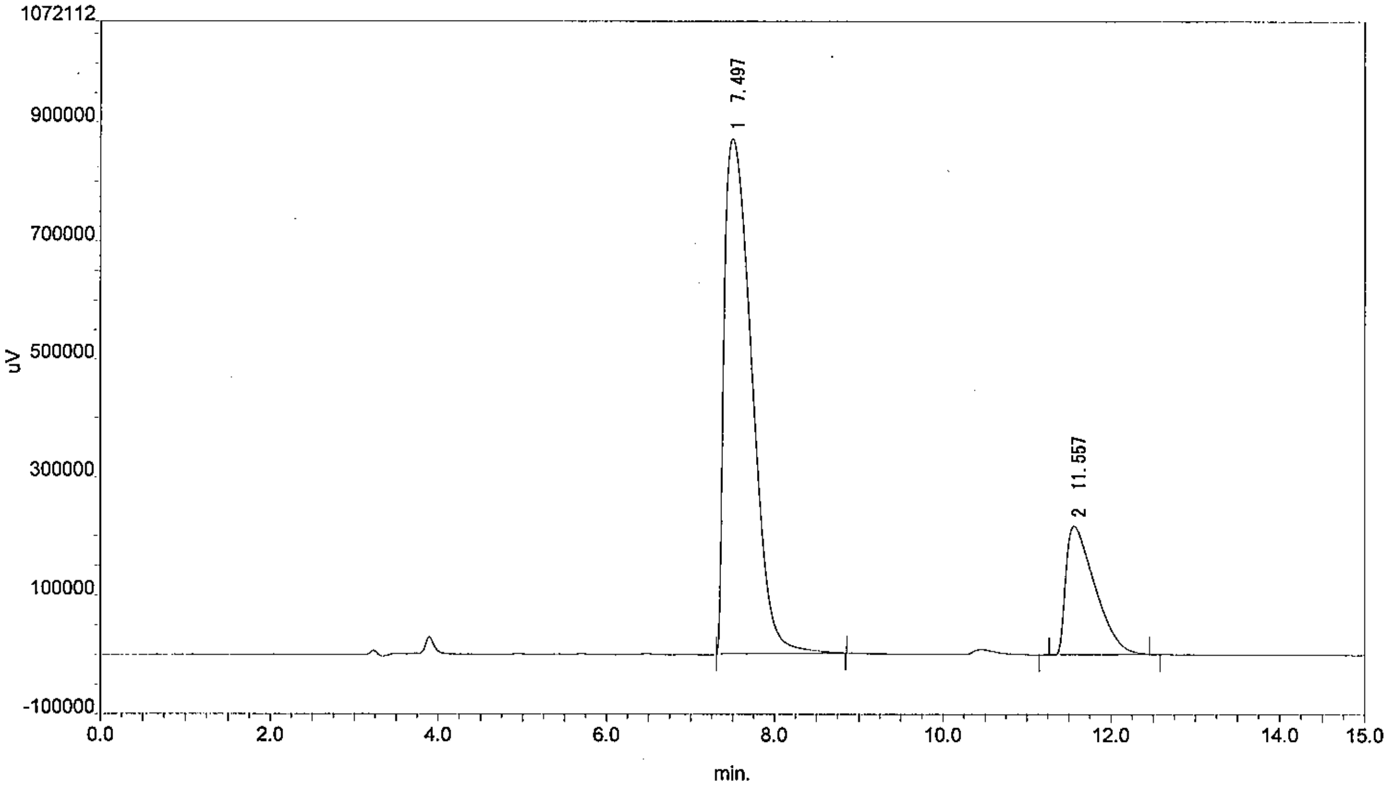

解析結果

No.	Rt(min)	ピーク名	面積	面積(%)	高さ	NTP	対称性	分離度
1	7.50		19769437.358	79.1388	871019	2871.3	2.128	6.726
2	11.56		5211277.354	20.8612	217207	5094.8	2.302	------
			24980714.712	100.0000	1088226			

Chromatogram Report

Table 5, Entry 5

分析条件

データファイル名	:	cl-Ph, 2-PrOH OD-3, 10%2017Y06M01D22h02m13s.crm
解析ファイル名	:	
チャンネルNo.	:	1
分析時間	:	30.0 min
取込間隔	:	200 msec
データ保存場所	:	c:¥users¥有機工業¥documents¥クロマトプロデータ¥yamazaki¥クロロ置換分割¥
分析日時	:	Thu Jun 01 22:02:13 2017
コメント	:	cl-Ph, 2-PrOH OD-3, 10%

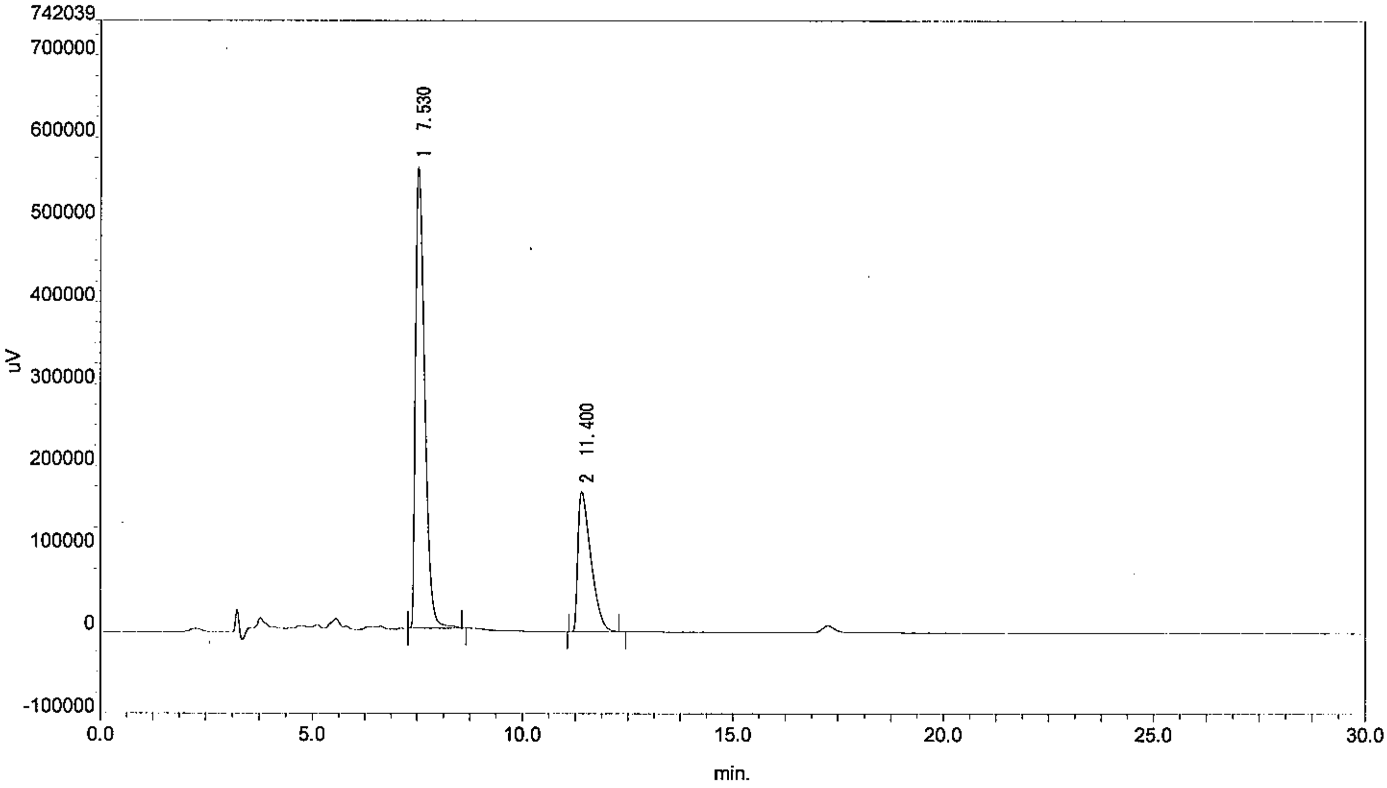

解析結果

No.	Rt(min)	ピーク名	面積	面積(%)	高さ	NTP	対称性	分離度
1	7.53		8502234.293	70.1863	560856	5718.1	1.837	7.869
2	11.40		3611576.417	29.8137	169236	6069.8	2.156	------
			12113810.709	100.0000	730092			

Chromatogram Report

Table 5, Entry 6

分析条件

データファイル名	:	cl-Ph, EtOH OD-3, 10%2017Y06M01D21h30m02s.crm
解析ファイル名	:	
チャンネルNo.	:	1
分析時間	:	12.8 min
取込間隔	:	200 msec
データ保存場所	:	c:¥users¥有機工業¥documents¥クロマトプロデータ¥yamazaki¥クロロ置換分割¥
分析日時	:	Thu Jun 01 21:30:02 2017
コメント	:	cl-Ph, EtOH OD-3, 10%

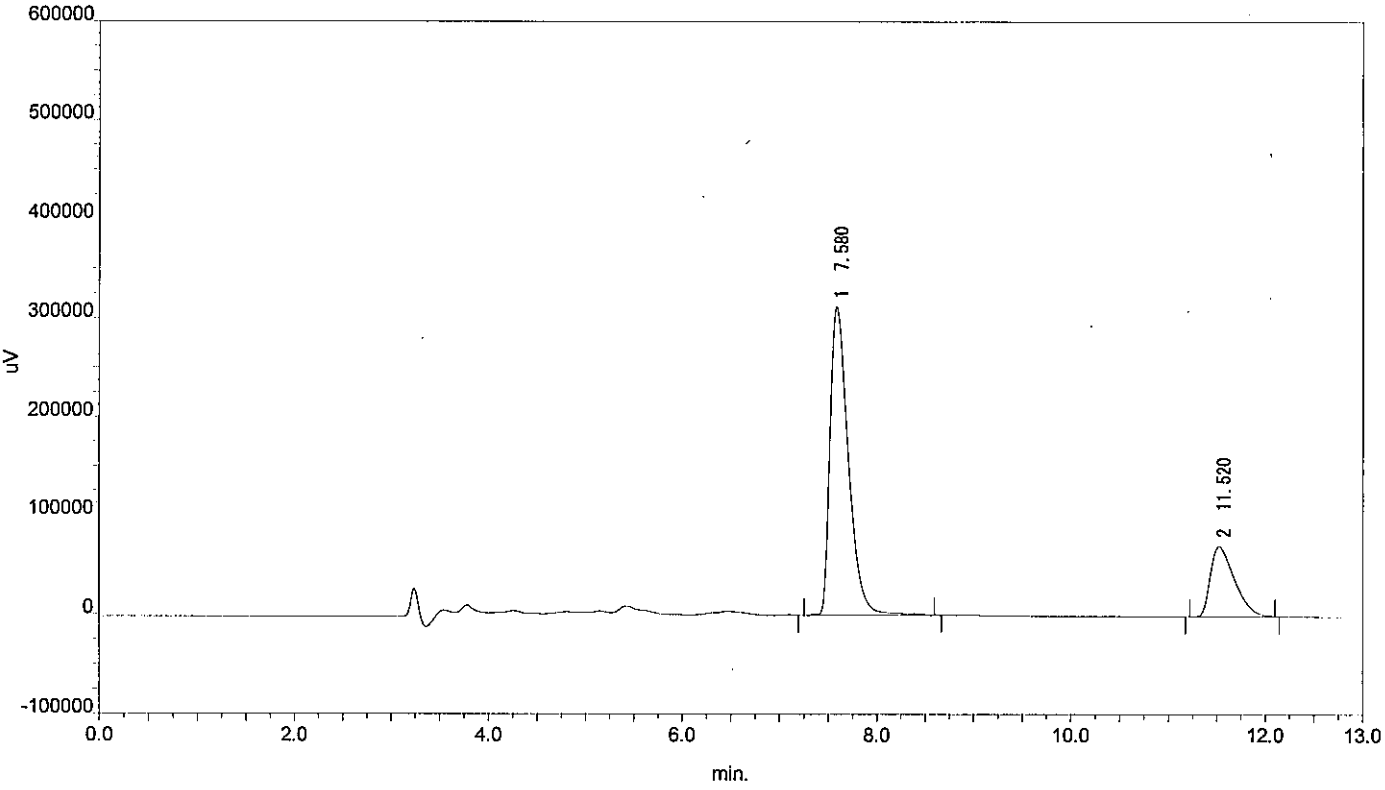

解析結果

No.	Rt(min)	ピーク名	面積	面積(%)	高さ	NTP	対称性	分離度
1	7.58		4101078.768	76.9813	311817	7549.7	1.673	9.609
2	11.52		1226293.900	23.0187	70568	9566.2	1.640	———
			5327372.668	100.0000	382385			

Chromatogram Report

Table 6, Entry 1

分析条件

データファイル名	:	3-OH-5-Ph.PA, 1,4-dioxane-1, 3.12.212021Y12M03D19h41m49s.crm
解析ファイル名	:	
チャンネルNo.	:	1
分析時間	:	20.0 min
取込間隔	:	200 msec
データ保存場所	:	c:¥documents and settings¥user¥my documents¥クロマトプロデータ¥srinivas¥3-oh-5-pl
分析日時	:	Fri Dec 03 19:41:49 2021
コメント	:	3-OH-5-Ph.PA, 1,4-dioxane-1, 3.12.21

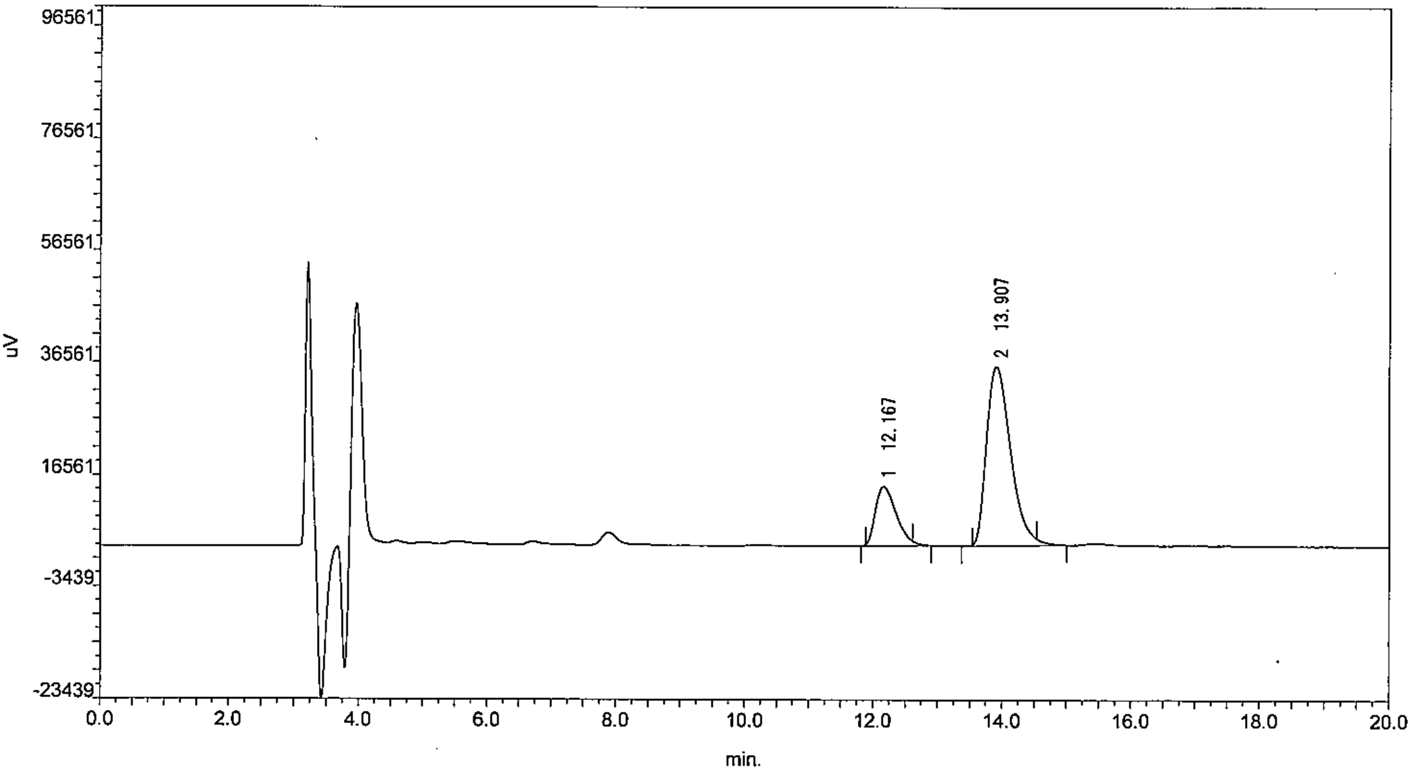

解析結果

No.	Rt(min)	ピーク名	面積	面積(%)	高さ	NTP	対称性	分離度
1	12.17		236695.106	22.2527	10626	6359.9	------	2.665
2	13.91		826971.680	77.7473	31974	6398.2	1.495	------
			1063666.786	100.0000	42600			

Chromatogram Report

Table 6, Entry 2

分析条件

データファイル名	:	3-OH-5-Ph.PA-1, toluene, 25.11.212021Y11M25D19h39m42s.crm
解析ファイル名	:	
チャンネルNo.	:	1
分析時間	:	25.0 min
取込間隔	:	200 msec
データ保存場所	:	c:¥documents and settings¥user¥my documents¥クロマトプロデータ¥srinivas¥3-oh-5-pl
分析日時	:	Thu Nov 25 19:39:42 2021
コメント	:	3-OH-5-Ph.PA-1, toluene, 25.11.21

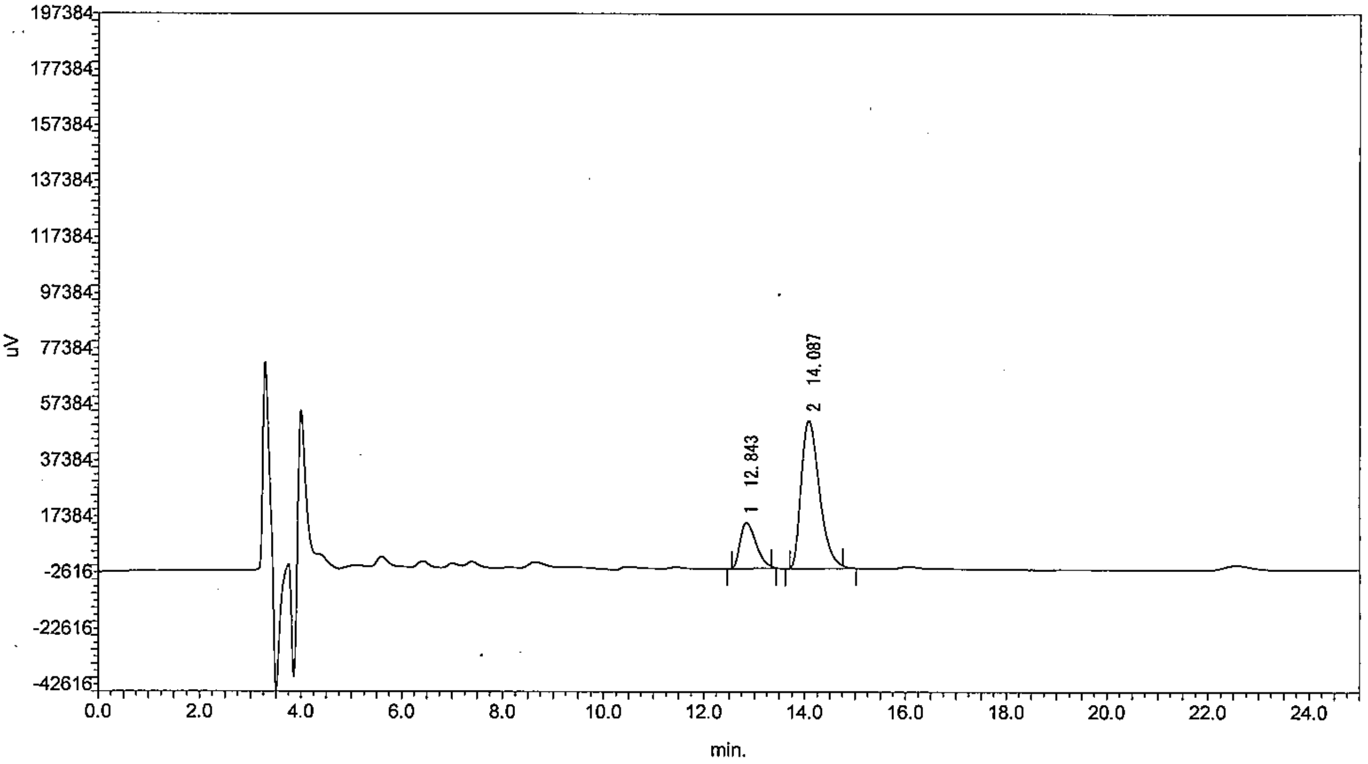

解析結果

No.	Rt(min)	ピーク名	面積	面積(%)	高さ	NTP	対称性	分離度
1	12.84		361811.637	21.3389	16281	7380.3	1.475	1.946
2	14.09		1333739.527	78.6611	52777	6864.9	1.404	------
			1695551.165	100.0000	69058			

Chromatogram Report

Table 6, Entry 3

分析条件
データファイル名　: 3-OH-5-Ph.PA, CHCl3-1, 2.12.212021Y12M02D23h07m32s.crm
解析ファイル名　:
チャンネルNo.　: 1
分析時間　: 20.0 min
取込間隔　: 200 msec
データ保存場所　: c:¥documents and settings¥user¥my documents¥クロマトプロデータ¥srinivas¥3-oh-5-pl
分析日時　: Thu Dec 02 23:07:32 2021
コメント　: 3-OH-5-Ph.PA, CHCl3-1, 2.12.21

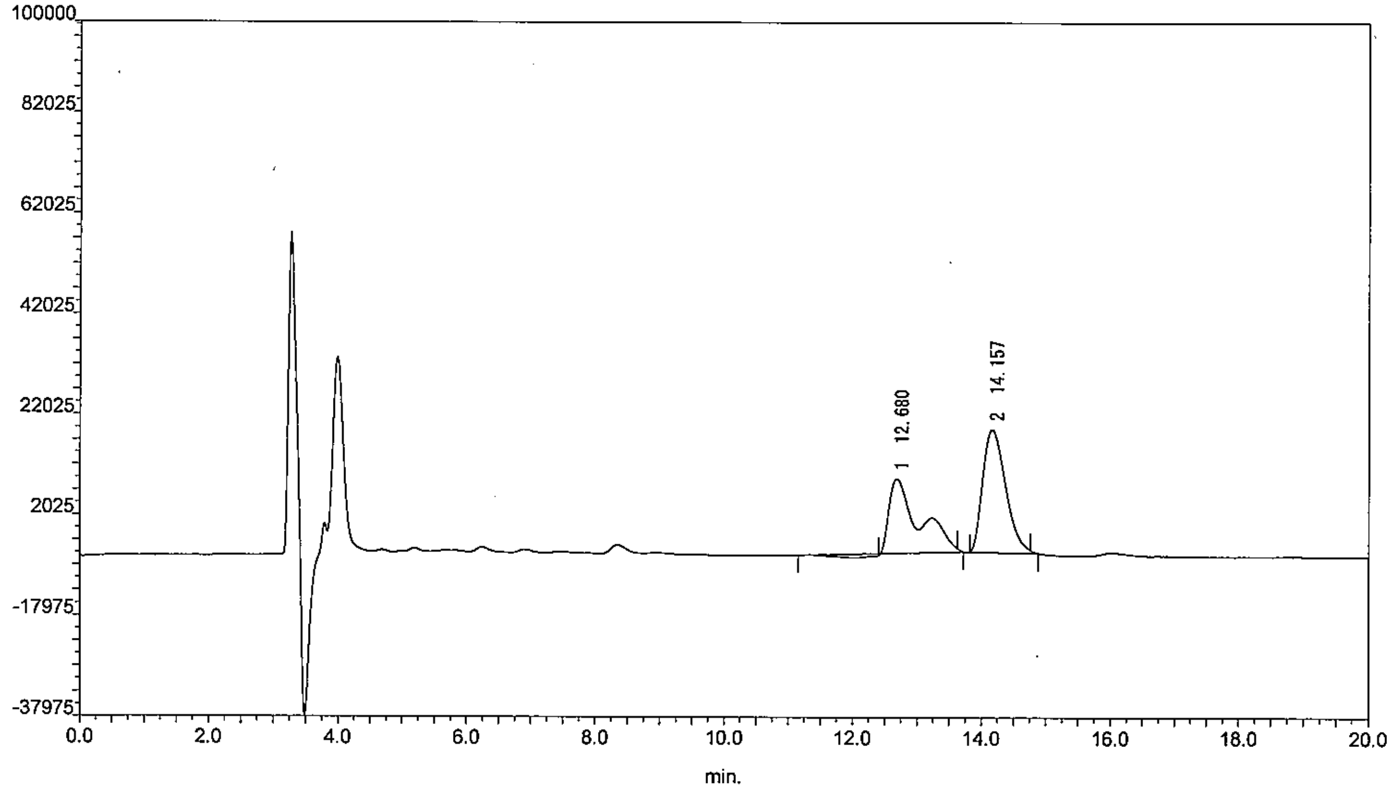

解析結果

No.	Rt(min)	ピーク名	面積	面積(%)	高さ	NTP	対称性	分離度
1	12.68		477678.532	44.2153	14793	2349.7	------	1.732
2	14.16		602667.547	55.7847	24470	7384.4	1.427	------
			1080346.079	100.0000	39263			

Chromatogram Report

Table 6, Entry 4

分析条件

データファイル名	: 3-OH-5-Ph.PA, ethylacetate, 17.12.212021Y12M17D22h35m58s.crm
解析ファイル名	:
チャンネルNo.	: 1
分析時間	: 20.4 min
取込間隔	: 200 msec
データ保存場所	: c:¥documents and settings¥user¥my documents¥クロマトプロデータ¥srinivas¥3-oh-5-pl
分析日時	: Fri Dec 17 22:35:58 2021
コメント	: 3-OH-5-Ph.PA, ethylacetate, 17.12.21

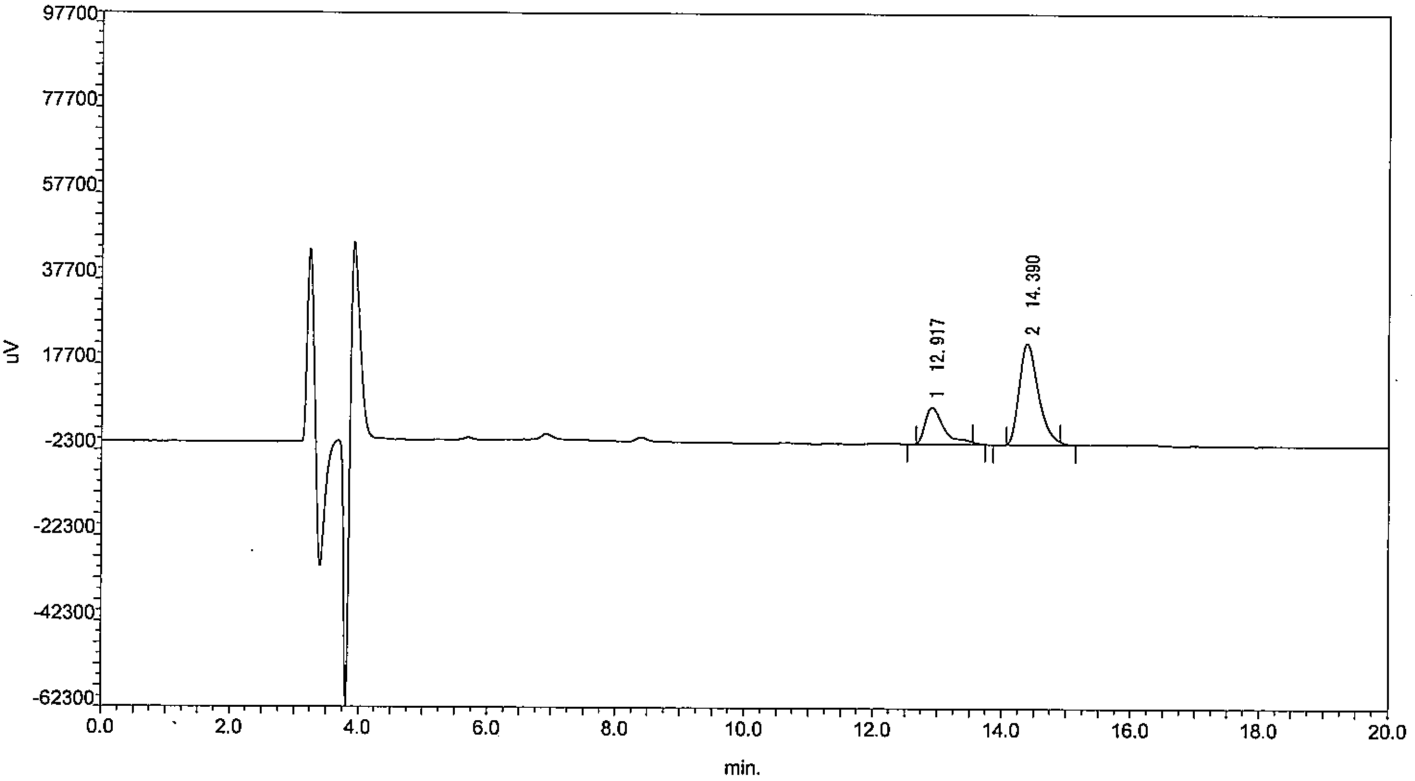

解析結果

No.	Rt(min)	ピーク名	面積	面積(%)	高さ	NTP	対称性	分離度
1	12.92		177159.628	26.4677	8573	5667.0	―――	2.330
2	14.39		492184.271	73.5323	23713	9905.7	1.389	―――
			669343.898	100.0000	32286			

Chromatogram Report

Table 6, Entry 5

分析条件
データファイル名 : 3-OH-5-Ph.PA, THF-1, 2.12.212021Y12M02D22h06m03s.crm
解析ファイル名 :
チャンネルNo. : 1
分析時間 : 20.0 min
取込間隔 : 200 msec
データ保存場所 : c:¥documents and settings¥user¥my documents¥クロマトプロデータ¥srinivas¥3-oh-5-pl
分析日時 : Thu Dec 02 22:06:03 2021
コメント : 3-OH-5-Ph.PA, THF-1, 2.12.21

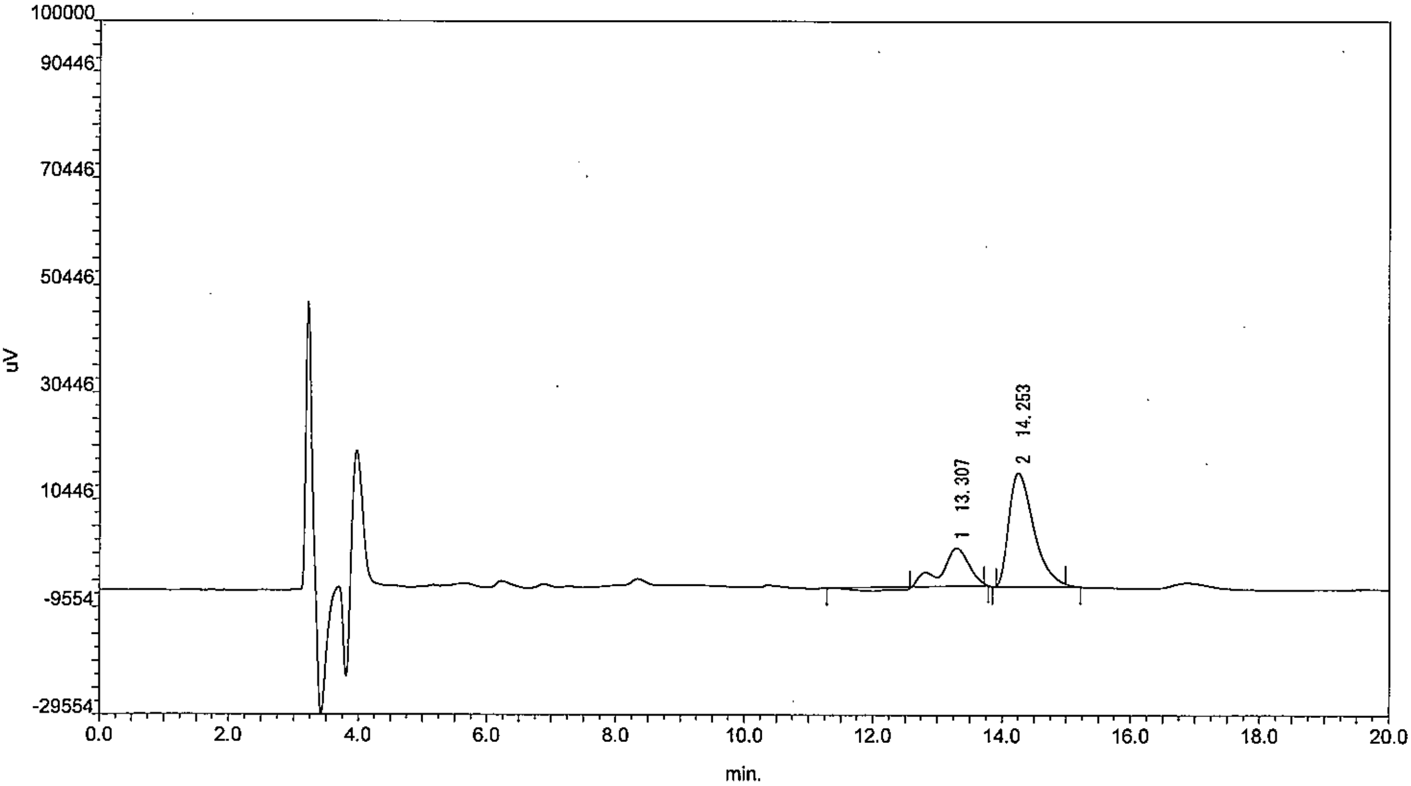

解析結果

No.	Rt(min)	ピーク名	面積	面積(%)	高さ	NTP	対称性	分離度
1	13.31		214697.050	27.3880	6990	2816.2	------	1.084
2	14.25		569211.204	72.6120	21141	5871.0	1.608	------
			783908.255	100.0000	28131			

Chromatogram Report

Table 6, Entry 6

分析条件

データファイル名	:	3-OH-5-Ph.PA, IPA-1, 9.11.212021Y11M09D21h55m59s.crm
解析ファイル名	:	
チャンネルNo.	:	1
分析時間	:	30.0 min
取込間隔	:	200 msec
データ保存場所	:	c:¥documents and settings¥user¥my documents¥クロマトプロデータ¥srinivas¥3-oh-5-pl
分析日時	:	Tue Nov 09 21:55:59 2021
コメント	:	3-OH-5-Ph.PA, IPA-1, 9.11.21

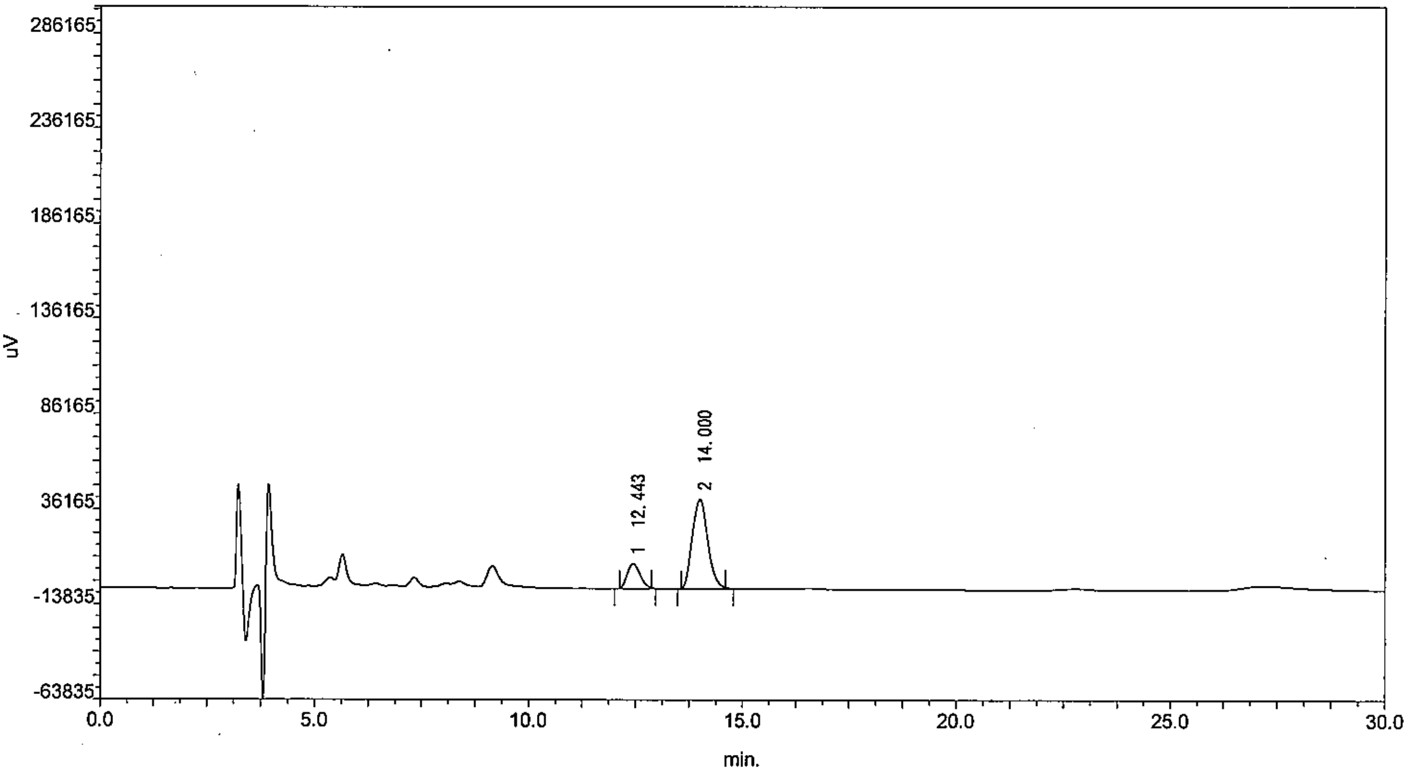

解析結果

No.	Rt(min)	ピーク名	面積	面積(%)	高さ	NTP	対称性	分離度
1	12.44		281412.585	19.1185	13175	7500.9	1.215	2.485
2	14.00		1190526.610	80.8815	46826	6823.4	1.178	------
			1471939.195	100.0000	60001			

Chromatogram Report

Table 6, Entry 8

分析条件

データファイル名	:	3-OH-5-Ph.PA, 50% EtOH, 20.12.212021Y12M20D18h28m09s.crm
解析ファイル名	:	
チャンネルNo.	:	1
分析時間	:	20.0 min
取込間隔	:	200 msec
データ保存場所	:	c:¥documents and settings¥user¥my documents¥クロマトプロデータ¥srinivas¥3-oh-5-pl
分析日時	:	Mon Dec 20 18:28:09 2021
コメント	:	3-OH-5-Ph.PA, 50% EtOH, 20.12.21

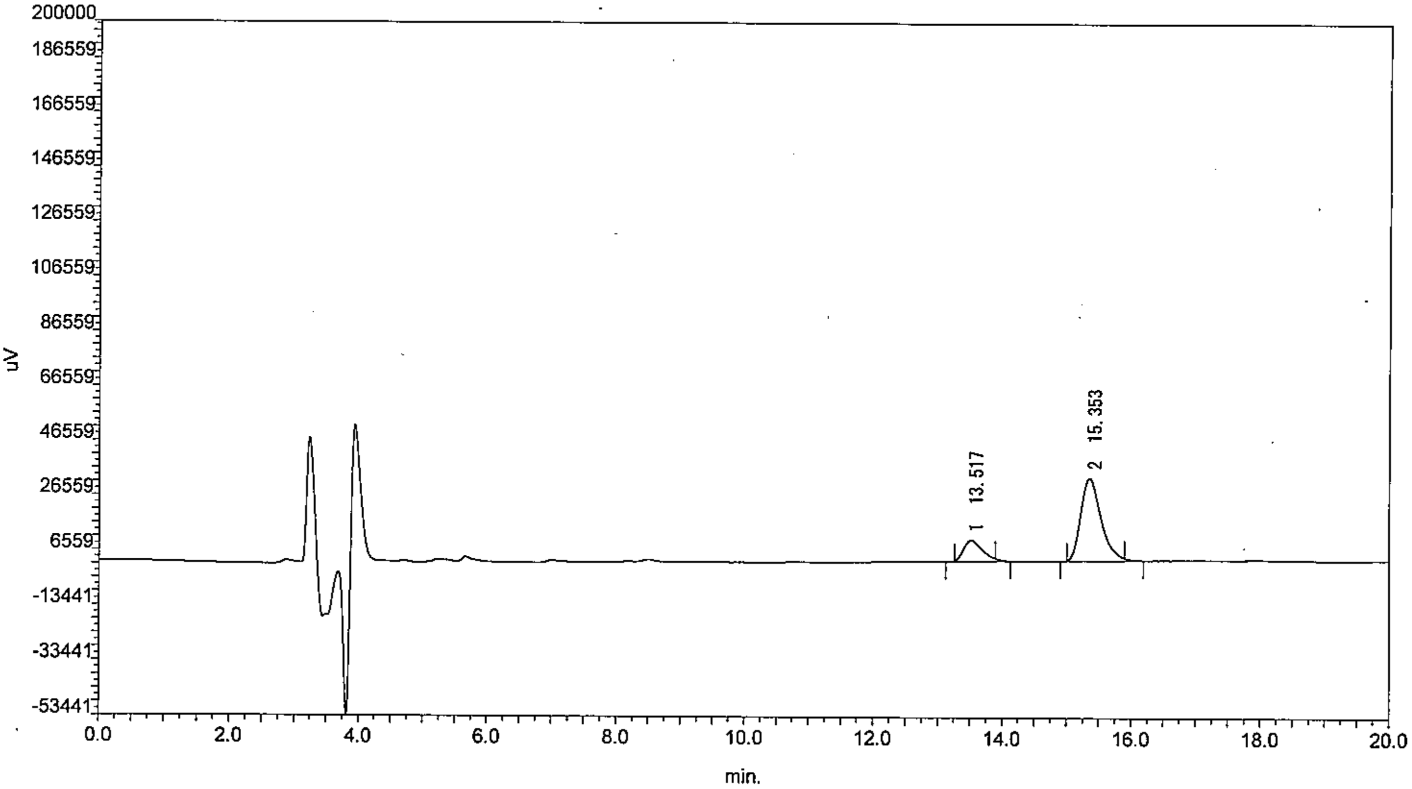

解析結果

No.	Rt(min)	ピーク名	面積	面積(%)	高さ	NTP	対称性	分離度
1	13.52		156146.747	19.0480	7695	8700.2	------	3.095
2	15.35		663608.916	80.9520	30254	10229.7	1.361	------
			819755.663	100.0000	37949			

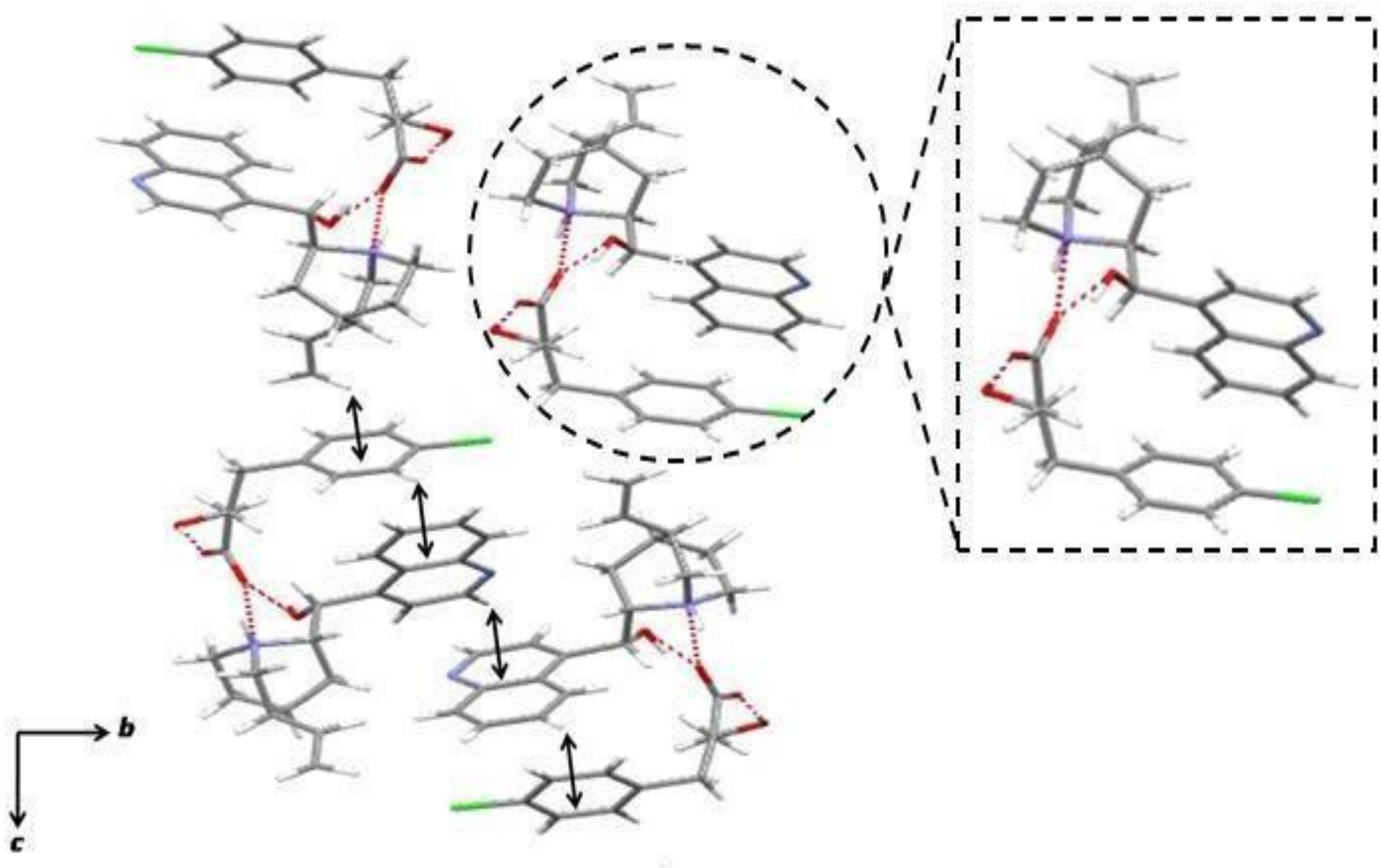

Figure S1. The (*R*)-**2** · cinchonidine salt obtained in an EtOH/toluene solution, viewed from the *a* axis. Thedotted lines and arrows indicate hydrogen bonds and CH/π interactions, respectively.

Table S1. Summary of crystallographic data reported in this study.

	(*S*)-**2** ▪ (+)-ADPE ▪ THF	(*R*)-**1** ▪ cinchonidine	(*R*)-**2** ▪ cinchonidine	(*R*)-**3** ▪ cinchonidine
empirical formula	$C_{28}H_{34}NO_5Cl$	$C_{29}H_{34}N_2O_4$	$C_{29}H_{33}N_2O_4Cl$	$C_{30}H_{36}N_2O_4$
formula weight	500.01	474.58	509.02	488.61
temperature (K)	150	150	150	150
crystal size (mm)	0.80 × 0.05 × 0.04	0.21 × 0.03 × 0.02	0.33 × 0.04 × 0.03	1.00 × 0.16 × 0.07
crystal system	monoclinic	orthorhombic	orthorhombic	orthorhombic
space group	$P2_1$	$P2_12_12_1$	$P2_12_12_1$	$P2_12_12_1$
a (Å)	14.011(3)	6.2815(10)	6.308(4)	6.4241(12)
b (Å)	5.7290(12)	15.074(2)	15.189(9)	15.084(3)
c (Å)	15.973(3)	26.456(4)	26.885(16)	26.364(5)
α (°)	90	90	90	90
β (°)	98.700(3)	90	90	90
γ (°)	90	90	90	90
V (Å^3)	1267.4(5)	2505.1(7)	2576(3)	2554.8(8)
Z	2	4	4	4
Dc (g/cm^3)	1.310	1.258	1.312	1.270
μ (Mo_{Ka}) (mm^{-1})	0.190	0.084	0.187	0.084
$\theta_{min/max}$ (°)	1.290/24.999	1.539/24.989	1.515/25.000	1.545/27.490
$R1$ [$F_0 > 2\sigma(F_0)$]	0.0620	0.0496	0.0528	0.0910
$wR2$ (all F_0^2)	0.1486	0.1237	0.1211	0.2461
GOF	0.963	0.684	0.679	1.067
measured reflns	6140	12041	12137	15052
independent reflns	4085	4410	4544	5841
observed reflns	2932	3664	2274	4294
reflns used	4085	4410	4544	5841
parameters	318	340	349	329
CCDC number	2173286	2173287	2173288	2173289
